# Immune landscape of a genetically engineered murine model of glioma compared with human glioma

**DOI:** 10.1172/jci.insight.148990

**Published:** 2022-06-22

**Authors:** Daniel B. Zamler, Takashi Shingu, Laura M. Kahn, Kristin Huntoon, Cynthia Kassab, Martina Ott, Katarzyna Tomczak, Jintan Liu, Yating Li, Ivy Lai, Rocio Zorilla-Veloz, Cassian Yee, Kunal Rai, Betty Y.S. Kim, Stephanie S. Watowich, Amy B. Heimberger, Giulio F. Draetta, Jian Hu

**Affiliations:** 1 Department of Genomic Medicine,; 2 Department of Cancer Biology, and; 3 UT Health Graduate School of Biomedical Sciences, The University of Texas MD Anderson Cancer Center, Houston, Texas, USA.; 4 Department of Immunology,; 5 Department of Neurosurgery,; 6 Department of Melanoma Medical Oncology, and; 7 Department of Neurological Surgery, Lou and Jean Malnati Brain Tumor Institute, Robert H. Lurie Comprehensive Cancer Center, Feinberg School of Medicine, Northwestern University, Chicago, Illinois, USA.

**Keywords:** Neuroscience, Oncology, Brain cancer, Cellular immune response, Mouse models

## Abstract

Novel therapeutic strategies targeting glioblastoma (GBM) often fail in the clinic, partly because preclinical models in which hypotheses are being tested do not recapitulate human disease. To address this challenge, we took advantage of our previously developed spontaneous *Qk/Trp53/Pten* (QPP) triple-knockout model of human GBM, comparing the immune microenvironment of QPP mice with that of patient-derived tumors to determine whether this model provides opportunity for gaining insights into tumor physiopathology and preclinical evaluation of therapeutic agents. Immune profiling analyses and single-cell sequencing of implanted and spontaneous tumors from QPP mice and from patients with glioma revealed intratumoral immune components that were predominantly myeloid cells (e.g., monocytes, macrophages, and microglia), with minor populations of T, B, and NK cells. When comparing spontaneous and implanted mouse samples, we found more neutrophils and T and NK cells in the implanted model. Neutrophils and T and NK cells were increased in abundance in samples derived from human high-grade glioma compared with those derived from low-grade glioma. Overall, our data demonstrate that our implanted and spontaneous QPP models recapitulate the immunosuppressive myeloid-dominant nature of the tumor microenvironment of human gliomas. Our model provides a suitable tool for investigating the complex immune compartment of gliomas.

## Graphical abstract

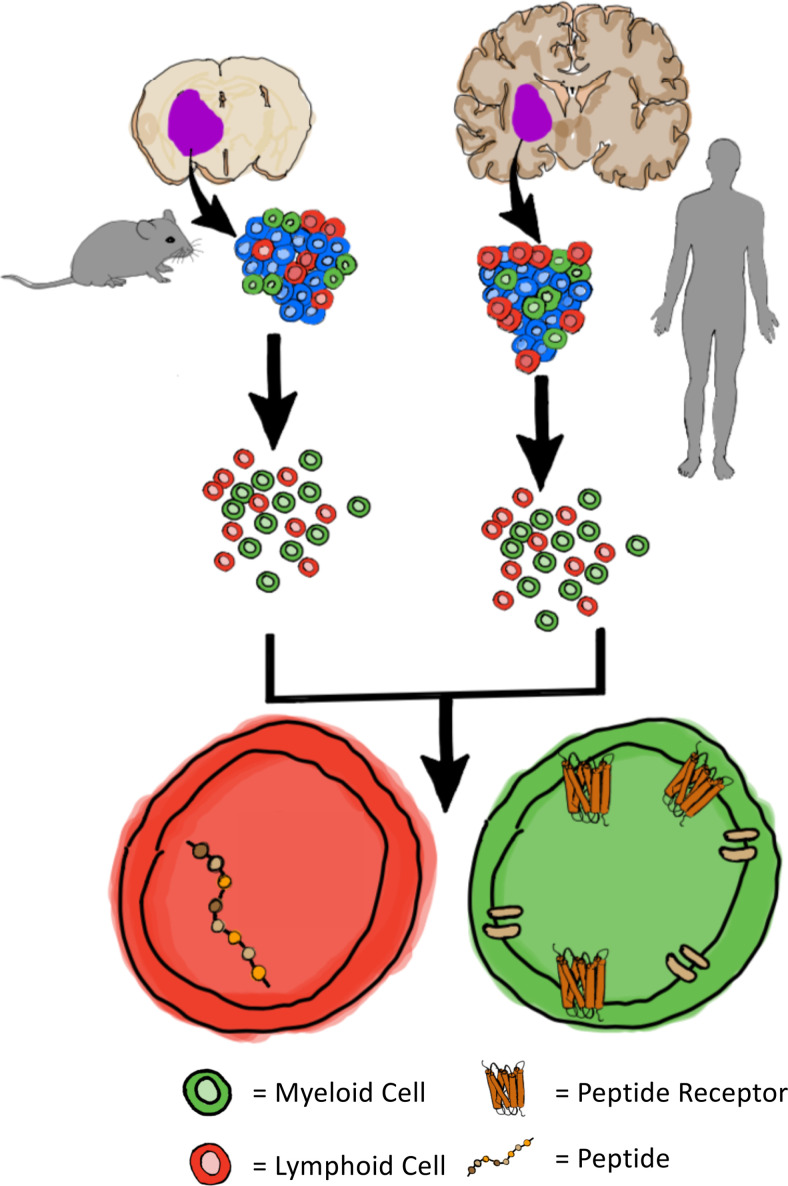

## Introduction

Efforts to advance novel drugs to treat the most frequent primary brain tumor, glioblastoma (GBM), have yet to substantially effect disease outcome (1). GBM thus remains the deadliest primary brain tumor, with a dismal median survival of only 15 months. Immunotherapies have substantially improved clinical outcomes in some indications, and patients with certain cancers previously recalcitrant to treatment have experienced disease remission or cure (2). Unfortunately, current immunotherapeutic strategies have not provided clinical benefit for most patients with GBM, probably owing to several factors, including T cell exhaustion and sequestration in the bone marrow, significant inter- and intrapatient heterogeneity, lack of synergy with standard-of-care treatment, low tumor mutational burden (TMB; 2.7/Mb average), and the presence of an overwhelming intratumoral immune-suppressive myeloid cell population (3–6). In GBM, tumor-associated myeloid cells (TAMs), which likely originate from both tissue-resident microglia and monocytes recruited from the peripheral circulation, are heterogeneous and can act in either an immunostimulatory or immunosuppressive capacity. The functions of the various TAM subpopulations remain unclear, and this issue is further confused by the lack of standardized nomenclature to describe TAM subpopulations. Notably, the TAM infiltrate of GBM remains understudied relative to infiltrating T cells in GBM, likely attributable to the success achieved by targeting T cells in other solid tumor types.

Currently, the most used preclinical model of GBM is the GL261 mouse glioma system. While tumors from GL261 mice can grow in syngeneic animals, compared with human GBM, these allograft tumors have low clonotypic diversity, are more highly antigenic, and have a higher TMB (~4978 mutations per Mb) (7). High TMB is associated with improved antitumoral efficacy of immune checkpoint blockade, and the high TMB in GL261 allografts might contribute to the antitumor efficacy of immune checkpoint blockade that has been observed using drugs that have then gone on to fail in human clinical trials (8).

To address the need for a preclinical model of GBM that more closely recapitulates the human tumor immune microenvironment, we characterized our immunocompetent murine spontaneous GBM model (9), which harbors deletion of 3 common tumor suppressor genes in human gliomas: quaking (*Qk* in mice and *QKI* in humans) (10), *Trp53* (11), and *Pten* (11). Termed QPP mice, this model uses an inducible cre-lox recombination system to delete the aforementioned genes under a nestin promoter that is expressed in neural stem cells, and QPP mice develop tumors with histopathological features of human GBM. The histopathological and transcriptomic heterogeneity observed among spontaneously arising tumors in QPP mice can manifest as any 1 of the 4 TCGA-described human GBM subtypes: proneural, classical, mesenchymal, or neural (9). This model and the original description of the histopathological features and heterogeneity of the tumor were originally described in Shingu et al. (9).

Originally identified as an RNA-binding protein (12), QKI also binds directly to DNA (13, 14), and its RNA/DNA binding activity promotes the transcription of genes involved in lipid metabolism in oligodendrocytes, lens epithelial cells, and microglia (13–16). *QKI* is mutated or deleted in approximately 34% of human GBMs (30% hemizygous deletions, 2% homozygous deletions, 2% mutations) (17). In addition, *QKI* downregulation by methylation of the *QKI* promoter was reported in 50 (20%) of 250 GBM samples (18). The significance of *QKI* deregulation in brain tumors is further highlighted by the observation that 90% of angiocentric gliomas, a type of low-grade pediatric glioma, contain *MYB-QKI* fusions, which transform cells by concomitantly activating *MYB* and suppressing *QKI* (19, 20).

Here, we characterize the immune microenvironments of spontaneous and allograft QPP tumors as well as patient-derived tumors. IHC and single-cell sequencing (scSeq) analyses of immune infiltrates determined that both spontaneous and allograft QPP tumors broadly recapitulated the intratumoral heterogeneity and interpatient variability of human gliomas. Overall, mouse and human tumors were infiltrated by similar immune cell populations, both regarding the type and proportion of immune cells present. Our findings indicate that QPP tumors are infiltrated by immune cells that broadly recapitulate the immune microenvironment of human GBM and that the QPP mice represent an important preclinical model to investigate the factors that influence the response of GBM to immunotherapeutics.

Patients with GBM currently have limited therapeutic options and a median survival time of only approximately 15 months (11). There is currently an unmet need for models of GBM that can accurately recapitulate human disease to improve the efficiency of translation of preclinical findings. Previously, we generated the QPP genetically engineered mouse model (GEMM) of GBM, which develops tumors with histological features similar to human GBM. Here, we demonstrate that both spontaneous and allograft QPP tumors recapitulate the composition and complexity of the human GBM immune infiltrate, which is predominantly myeloid, with T, B, and NK cell components. Our study validates QPP tumors as a crucial preclinical tool to investigate tumor-immune crosstalk that might identify clinically effective approaches to target the immune microenvironment of GBM.

## Results

### Comparison of the QPP models with GBM.

We characterized immune infiltrates in spontaneous QPP tumors arising in GEMMs as well as in QPP-derived tumors implanted into syngeneic host animals. For the latter, we prioritized our most widely distributed QPP tumor cell line model, QPP7 (Supplemental Figure 1A; supplemental material available online with this article; https://doi.org/10.1172/jci.insight.148990DS1), which we confirmed generates tumors with near-complete penetrance upon orthotopic implantation into the striatum of C57BL/6J mice (Supplemental Figure 1E). Both spontaneous and implanted QPP tumors displayed key histopathological features of human GBM, including necrotic areas (Supplemental Figure 1, B and F), invasive leading edges (Supplemental Figure 1, C and G), and a high proliferative index, as assessed by Ki67 staining (Supplemental Figure 1, D and H). GEMM QPP mice developed GBM with 100% penetrance and had a median survival time of approximately 90 days, consistent with previous findings (9). By comparison, mice with QPP7-derived allograft tumors had a median survival time of approximately 45 days, which is indicative of more aggressive disease (Supplemental Figure 2A).

To assess the TMB of the 2 models, we collected matched tumor and tail tissue from 5 QPP GEMM mice, and we analyzed tissue derived directly from the spontaneous tumor from which the QPP7 cell line was generated. All tumors used in this analysis harbored the wild-type *Idh1* allele. Isolated DNA was multiplexed and sequenced, and we used the standard gatk pipeline to call variants according to the MuTect filters (21–24). TMB values varied among the spontaneous tumors (22.225, 49.225, 8.375, 386.9, and 4.875 mutations/Mb), and in the QPP7 tumors, the TMB was 2.775 (Supplemental Figure 2B). By comparison, the reported TMB for GL261 allografts is 4978 mutations/Mb, which is order of magnitude higher than the average human GBM TMB of 2.7 mutations/Mb (5).

### Comparison of the immune infiltrates of QPP models with glioma.

**Figure 1:**
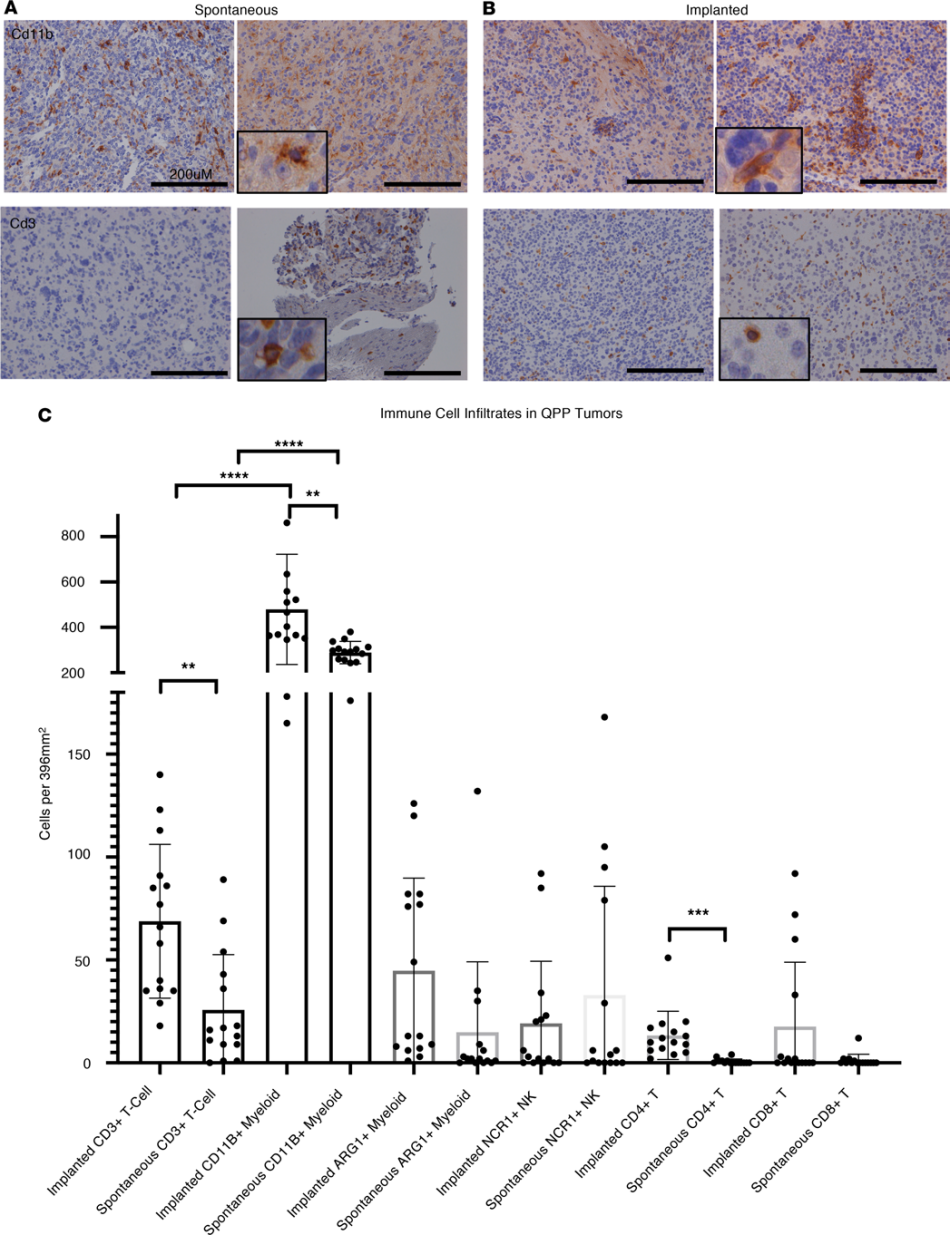
IHC of immune markers. (**A**) Staining showing representative images of CD11B and CD3^+^ cells in the brains of mice with spontaneous QPP tumors. (**B**) Staining showing representative images of CD11B and CD3^+^ cells in the brains of mice with implanted QPP tumors. (**C**) Three separate fields from *n* = 5 mice were stained with CD3 for pan T cells; lysosomal-associated membrane protein 1 (LAMP1) for myeloid cells; arginase-1 (ARG1) for tumor-associated myeloid cells; NK cell receptor 1 (NCR1) for NK cells; CD4 for T helper cells; and CD8 for cytotoxic T cell lymphocytes. Original magnification, ×20; ×60 (inset images). Scale bars: 200 μM. Statistics were performed using an unpaired *t* test with Welch’s correction (2 tailed). **P < 0.01; ***P < 0.001; ****P <0.0001.

Next, IHC analysis was performed on QPP spontaneous and QPP7 allograft tumors (*n* = 5 tumors each) and on patient-derived tumor samples (*n* = 5 samples) using antibodies against major immune constituents. Characteristics of the mouse and human samples are provided in Supplemental Table 1, and the antibodies used are listed in Supplemental Table 2. The immune infiltrate was similar between both spontaneous and implanted QPP tumors as well as between both mouse models and the human tumor samples. Specifically, myeloid and lymphoid infiltrates, including T and NK cells, were detected in all 3 data sets (antibodies are listed in Supplemental Table 2), and the pervasiveness and intensity of myeloid cell markers, including CD11B and arginase 1, were much higher compared with lymphoid markers in all 3 tumor models (Figure 1 and Supplemental Figure 3, A and B). Only CD11B, which is expressed on tissue-resident microglia, was detected in nontumor-bearing control brain tissues isolated from C57BL/6J mice (*n* = 5 samples; data not shown), confirming that these immune cell populations are restricted to the pathogenic brain. These findings align with previous characterizations of GBM tumors (6, 25). Quantification of the stains showed that, in both the spontaneous and implanted QPP models, there were significantly more myeloid cells than T cells. In addition, both myeloid and T cell infiltrates were more abundant in the implanted versus spontaneous tumors, and the immune infiltrates were similar to those from the human tumor tissue (Supplemental Figure 3C).

Approaches such as IHC use single markers or limited marker profiles to define cell populations, but these techniques may be unable to account for all differences that may exist in the immune cell populations from diverse species (26–28). We used scSEQ to delineate and compare the specific immune cell populations in spontaneous QPP, QPP7 allograft, and human tumor tissues. Freshly isolated or previously frozen samples from mouse or human GBM tissue were processed using the 10x Genomics scSEQ platform. Batch-corrected cohorts of CD45^+^-enriched immune cells from spontaneous (*n* = 3) and implanted (*n* = 3) QPP tumors, as well as from the same cohort of human tumors (*n* = 15), were analyzed. Although small compared with other GBM data sets (11), our analyses were conducted on purified immune cells, whereas previous studies have conducted ancillary analyses of immune cells in a mixed population with tumor cells. Our analysis showed that the myeloid and lymphoid constituents were similar between batch-corrected CD45^+^ cells isolated from fresh compared with frozen tumor-free mouse brains (Supplemental Figure 4), indicating that there was no effect of storage on these cell populations.

**Figure 2:**
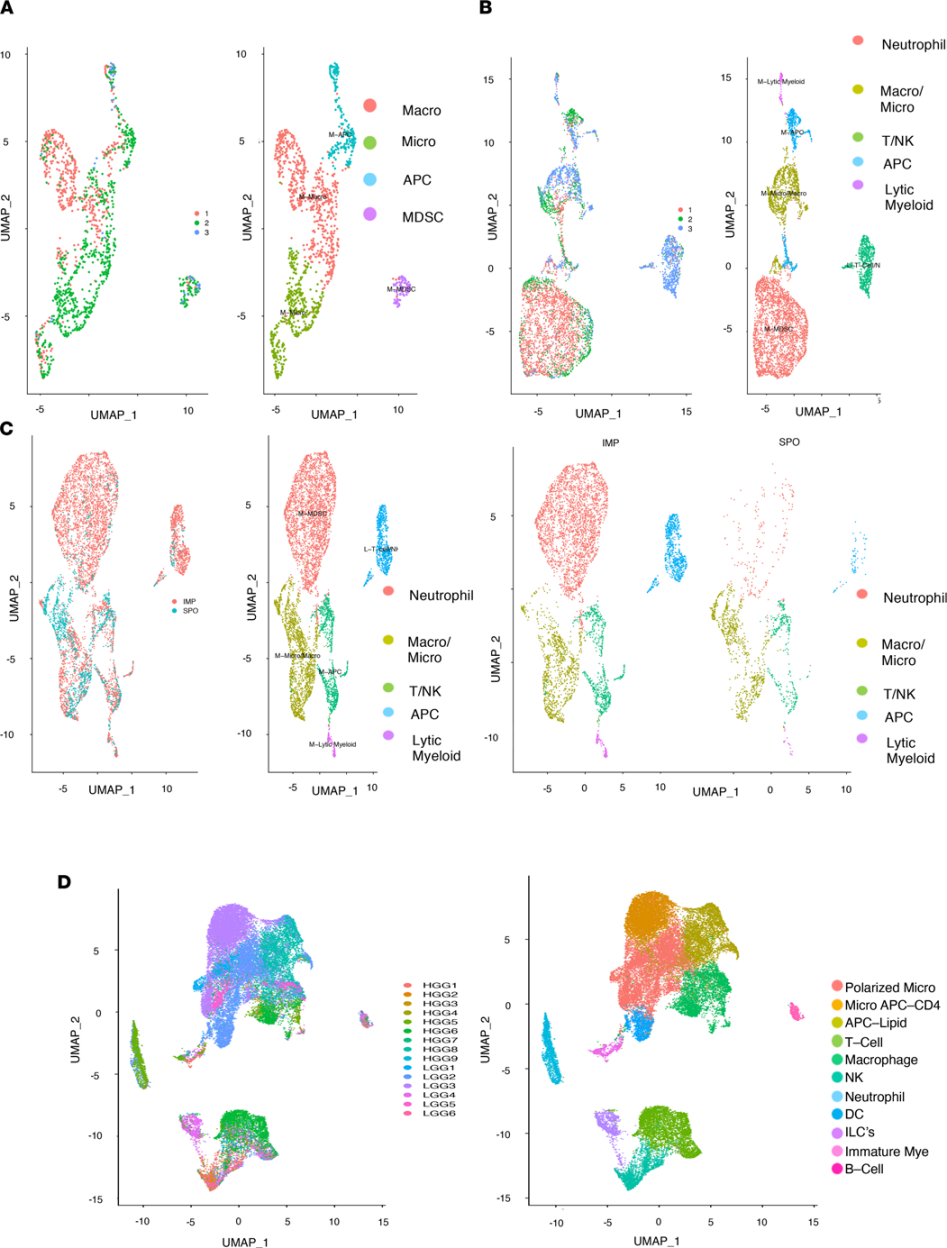
Clustering identifies major immune constituents. (**A**) UMAP clustering at a resolution of 0.1 of immune constituents from *n* = 3 spontaneous QPP mice, showing macrophages, microglia, antigen-presenting-like cells (APCs), and neutrophils with 9 principal components (PCs), determined by elbow plot. (**B**) UMAPs of immune constituents from *n* = 3 implanted QPP7 mice, showing neutrophils, microglia/macrophages, T cells/NK cells, APCs, and lytic myeloid cells with 9 PCs, determined by elbow plot. (**C**) Comparison of the implanted and spontaneous QPP tumor clusters showing neutrophils, microglia/macrophages, T cells/NK cells, APCs, and lytic myeloid cells with 9 PCs, determined by elbow plot. (**D**) UMAPs of immune constituents from *n* = 15 patients with glioma, showing clusters of polarized microglia, APC microglia, APCs, T cells, macrophages, NK cells, neutrophils, DCs, ILCs, immature myeloid cells, and B cells with 20 PCs, determined by elbow plot.

scSeq analysis of spontaneous QPP tumors demonstrated that the majority of intratumoral immune cells were myeloid (Supplemental Figure 5), and 4 subtypes were identified (Figure 2A): macrophages, microglia (Supplemental Figure 6B), neutrophils (Supplemental Figure 6C), and myeloid antigen-presenting-like cells (APCs; Supplemental Figure 6D). T, B, and NK cells were also detected in these samples, but their levels were insufficient for subtype cluster analysis (Supplemental Figure 6, E–G). All clusters were defined on the basis of the 3 representative markers shown in Supplemental Figure 6 as well as using a variety of other markers selected from the top differentially expressed genes for that cluster. The full list of genes upregulated for each cluster is presented in Supplemental Table 3.

In the data set from implanted QPP7 tumors (Supplemental Figure 7), scSEQ analysis identified 5 clusters (Figure 2B): microglia/macrophages (Supplemental Figure 8B), neutrophils (Supplemental Figure 8C), APCs (Supplemental Figure 8D), lytic myeloid cells (Supplemental Figure 8E), and T, B, and NK cells (Supplemental Figure 8, F–H). The full list of genes upregulated for each cluster can be found in Supplemental Table 4. These cell populations are consistent with our IHC analysis in both spontaneous and implanted QPP mouse tumors and are also congruent with reported human data (29). The heterogeneity observed in the immune cell compartment among QPP7 allograft tumors is consistent with the macroscopic heterogeneity of the tumors when implanted as well as with the genomic instability described in ref. 9. Representative images of tumor slices from the implanted QPP7 line can be found in Supplemental Figure 9.

We next combined the scSEQ data from the spontaneous and allograft tumors as a single data set (Figure 2C and Supplemental Figure 10). Five major clusters were identified: neutrophils (Supplemental Figure 11B), microglia/macrophages (Supplemental Figure 11C), myeloid APCs (Supplemental Figure 11D), lytic myeloid cells (Supplemental Figure 11E), and T and NK cells. A population of B cells was also present, although these cells did not cluster (Supplemental Figure 11, F–H). The full list of genes upregulated for each cluster can be found in Supplemental Table 5.

Notably, our comparison uncovered a higher proportion of neutrophil, T cell, and NK cell infiltrates in QPP7 allograft tumors compared with spontaneous QPP tumors. We also observed slight variations among different mice in both the spontaneous QPP and implanted QPP7 cohorts; specifically, some mice in each cohort had more neutrophils than others (Supplemental Figure 12, A–C). This finding is consistent with the heterogeneous nature of the human disease. Importantly, the observed heterogeneity was not caused by technical variations, which were found to be minimal by examining 2 technical replicates (data not shown).

scSEQ analysis of the cohort of human glioma samples (resolution = 0.15; Supplemental Figure 13) identified 11 clusters (Figure 2D): polarized microglia (Supplemental Figure 14B); micro-APCs (CD4) and APCs (lipid) (Supplemental Figure 14C); T cells (Supplemental Figure 14D); macrophages and NK cells (Supplemental Figure 14E); DCs (Supplemental Figure 14F); neutrophils (Supplemental Figure 14G); and ILCs, immature myeloid cells, and B cells (Supplemental Figure 14H). The full list of genes upregulated for each cluster is presented in Supplemental Table 6. The intratumoral immune infiltrates were similar across all samples and were largely dominated by myeloid populations with minor lymphoid components (Supplemental Figure 14). Microglia and macrophages were present across all samples (Supplemental Figure 12C). Furthermore, the dominant intratumoral immune cell populations identified by scSEQ (Figure 2) aligned with our IHC analysis (Figure 1), and they were also congruent with those reported by other human GBM studies (29). Taken together, our scSEQ analysis demonstrated that both spontaneous QPP and QPP7 allograft tumors harbor immune cell populations that are similar to the infiltrates observed in human GBM.

We next compared the immune infiltrate of our spontaneous and implanted QPP models with human high- and low-grade glioma (HGG and LGG, respectively). Our 15-sample human tumor cohort included 9 histopathologically diagnosed HGG tumor samples and 6 LGG tumors samples (2 grade II oligodendroglioma and 4 grade II diffuse astrocytoma; Supplemental Table 2). When comparing the main immune constituents of LGG and HGG tumors, we observed a shift from a more microglial signature to a more macrophage-like signature, respectively. Similar to the mouse allograft tumors, higher-grade human tumors were characterized by an increase in the number of lymphoid infiltrates as well as an increase in infiltrating neutrophils compared with the spontaneous mouse tumors or human LGG tumors, respectively (Supplemental Figure 15). Even though these differences warrant further investigation, none of these differences effect the overall conclusion that the QPP mouse and allograft tumors represent improved preclinical models to study GBM.

**Figure 3:**
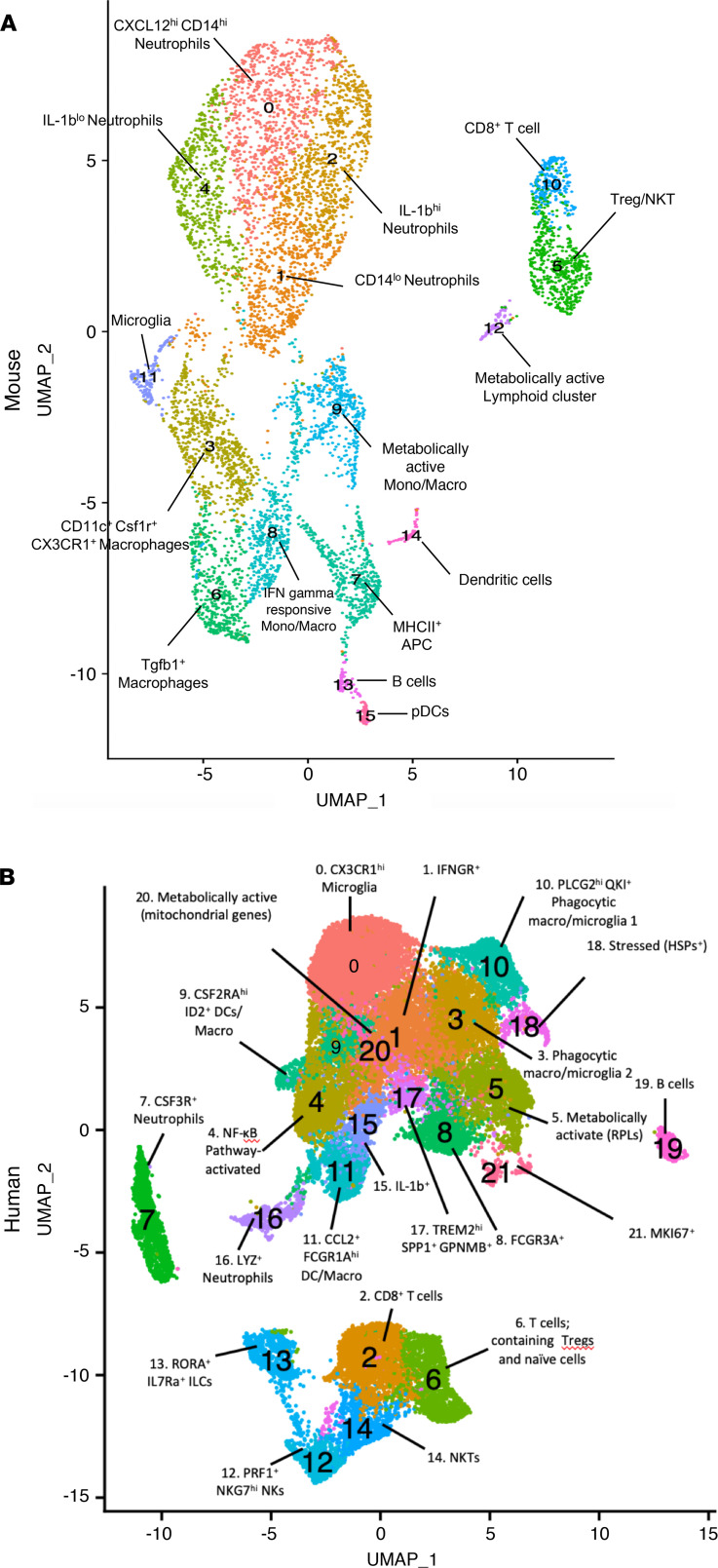
Clustering to reveal subtypes. (**A**) Subtype clustering at resolutions of 0.65 and 0.8 combined mouse spontaneous and implanted QPP tumor data set with 9 PCs determined by elbow plot. (**B**) Aggregated human GBM data set with 20 PCs determined by elbow plot.

We next performed subtype clustering to determine the minor immune constituents (resolution = 0.65) and to identify subpopulations among the major immune cell clusters (Figure 3) in mouse and human tumors. In our combined mouse tumor data set, we found populations of Cxcl12^hi^Cd14^hi^ neutrophils, Cd14^lo^ neutrophils, Il1-b^hi^ neutrophils, Cd11c^+^Csf1r^+^Cx3cr1^+^ macrophages, Il-1b^lo^ neutrophils, Tregs/NK T cells, Tgfb1^+^ macrophages, MHCII^+^ APCs, Ifn-γ–responsive monocytes/macrophages, metabolically active monocytes/macrophages, Cd8^+^ T cells, microglia, metabolically active lymphoid, B cells, DCs, and pDCs. In the human glioma samples, we found populations of CX3CR1^hi^ microglia, IFNGR^+^ microglia, CD8^+^ T cells, phagocytic microglia/macrophages, NF-κB pathway–activated myeloid cells, protein-producing myeloid cells, Tregs, naive T cells, CSF3R^+^ neutrophils, FCGR3A^+^ myeloid cells, CSF2RA^hi^ ID2^+^ DCs/macrophages, PLCG2^hi^ QKI^+^ phagocytic microglia/macrophages, CCL2^+^FCGR1A^hi^ DCs/macrophages, PRF1^+^NKG7^hi^ NK cells, RORA^+^IL7Ra^+^ ILCs, NK T cells, IL1b^+^ myeloid cells, LYZ^+^ neutrophils, TREM2^hi^SPP1^+^GPNMB^+^ myeloid cells, stressed (HSP^+^) B cells, metabolically active myeloid cells, and proliferating myeloid cells. The full lists of genes upregulated for each cluster is presented in Supplemental Table 7 (mice) and Supplemental Table 8 (human).

**Figure 4:**
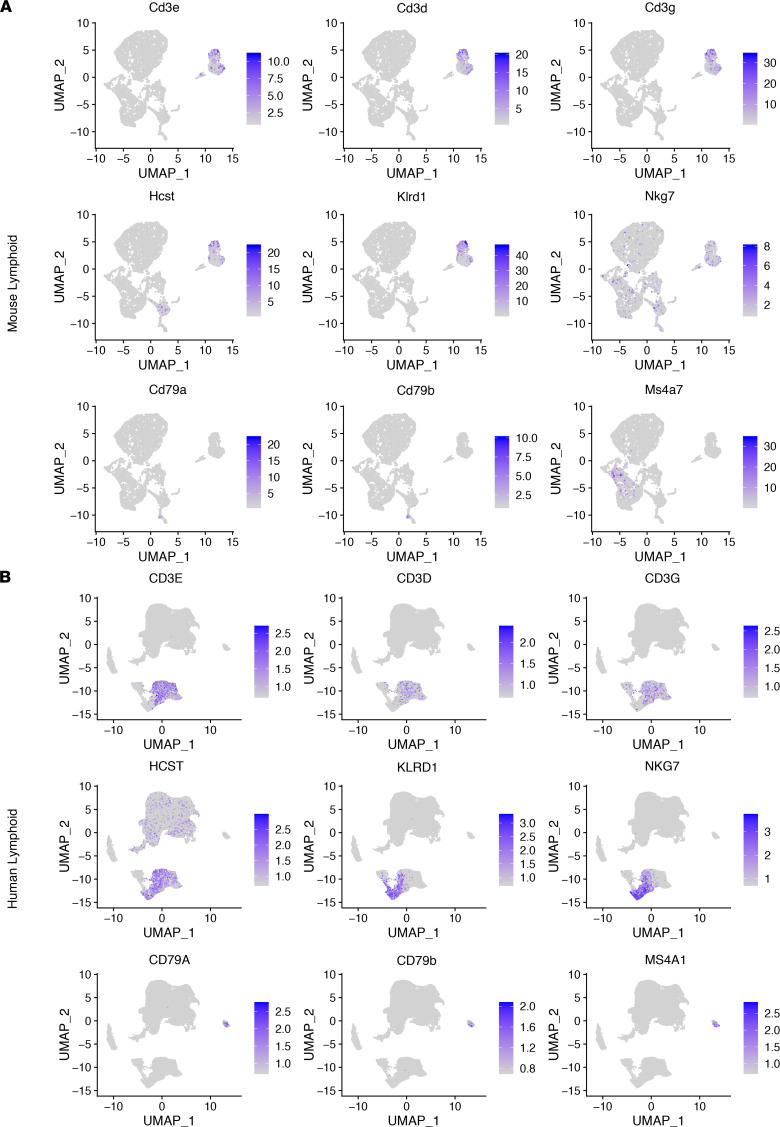
Lymphoid cell subtypes of human GBM are identified in the QPP model. Clustering at a resolution of 0.65 to reveal lymphoid subtypes in the QPP mouse model tumor or human GBM. UMAPs of immune constituents from (**A**) QPP-derived tumor (*n* = 6) and (**B**) patient glioma (*n* = 15) samples, showing clusters of lymphoid subtypes and representative markers for the following populations: T cells (Cd3d, Cd3e, and Cd3g as well as CD3D, CD3E, and CD3G); NK cells (Klrd1, Nkg7, and Nktr as well as KLRD1, NKG7, and NKTR; and B cells (Cd79a, Cd79b, and Ms4a7 as well as CD79A, CD79B, and MS4A7.

**Figure 5:**
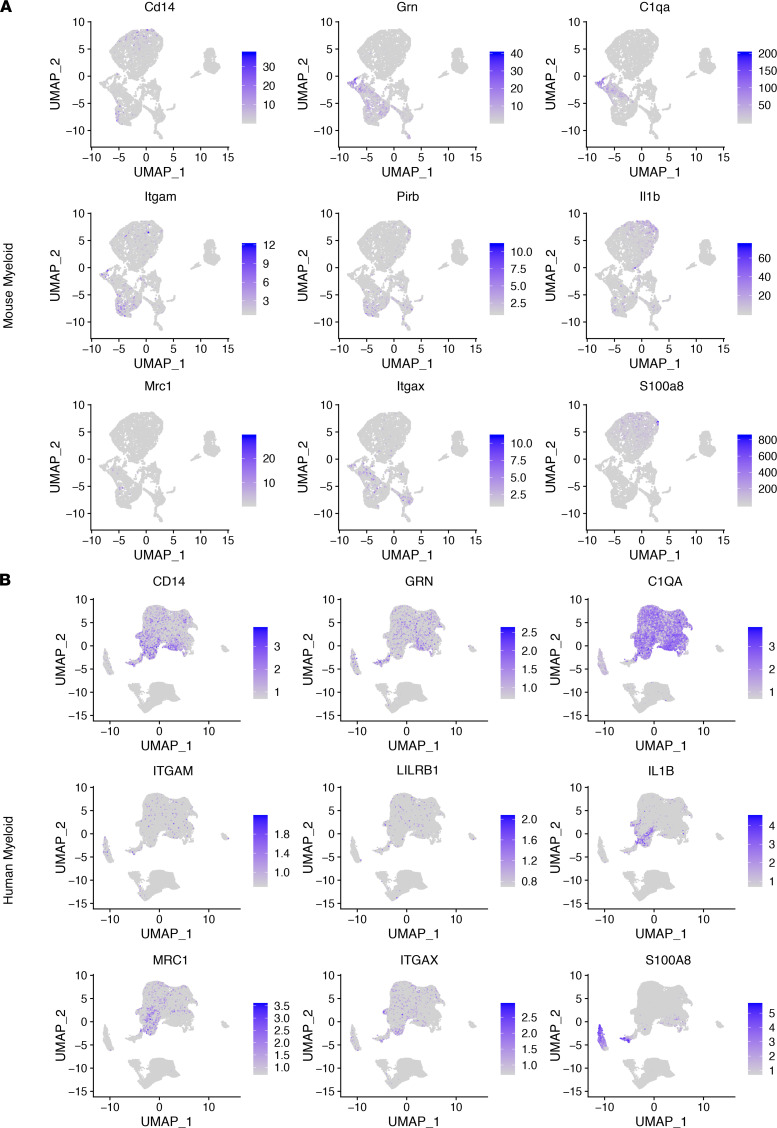
Myeloid cell subtypes of human GBM are identified in the QPP model. Clustering at a resolution of 0.65 to reveal myeloid subtypes in the QPP mouse model tumor or human glioma. UMAPs of immune constituents from (**A**) QPP-derived tumor (*n* = 6) and (**B**) patient glioma (*n* = 15) samples showing clusters of myeloid subtypes and representative markers for the following populations: monocytes (Cd14 and CD14), microglia (Grn and GRN), complement-expressing microglia (C1qa and C1QA), macrophages (Itgam and ITGAM), M0-like macrophages (Pirb and LILRB1), M1-like macrophages (Il1b and IL1B), M2-like macrophages (Mrc1 and MRC1), DCs (Itgax and ITGAX), and myeloid-derived suppressor cells (S100a8 and S100A8). Given the spectral nature of myeloid differentiation and polarization many of these markers will be present in multiple subsets.

We identified consistency among all 3 data sets (mouse implanted, mouse spontaneous, and human samples) within the lymphoid compartment, which include T cells that were marked by *Cd3d*, *Cd3e*, and *Cd3g* in mouse data sets (*CD3D*, *CD3E*, and *CD3G* in human data sets), NK cells that are marked by *Klrd1*, *Nkg7*, and *Nktr* (*KLRD1*, *NKG7*, and *NKTR* in human data sets), and B cells that are marked by *Cd79a*, *Cd79b*, and *Ms4a7* (*CD79A*, *CD79B*, and *MS4A7* in human data sets) (Figure 4). With regard to myeloid cells, we used the following markers: *Cd14* in mouse data sets (*CD14* in human data sets) for monocytes, *Grn* (*GRN* in human data sets) for microglia, *C1qa* (*C1QA* in human data sets) for complement-expressing microglia, *Itgam* (*ITGAM* in human data sets) for macrophages, *Pirb* (*LILRB1* in human data sets) for M0-like macrophages, *Il1b* (*IL1B* in human data sets) for M1-like macrophages, *Mrc1* (*MRC1* in human data sets) for M2-like macrophages, *Itgax* (*ITGAX* in human data sets) for DCs, and *S100a8* (*S100A8* in human data sets) for myeloid-derived suppressor cells. Similar with lymphoid subpopulations, each of these myeloid subtypes was detected in both QPP spontaneous and allograft mouse data sets as well as in the human data set. Further, given the spectrum nature of myeloid cell polarization, many of these markers were predictably identified across multiple myeloid subtypes (Figure 5). The number of cells that passed QC for each sample is available in Supplemental Table 9. Taken together, single-cell analysis of the immune infiltrates of murine and human GBM tissues was consistent with our histopathological analysis. Our findings support the hypothesis that tumors derived from both spontaneous and allograft QPP tumor models faithfully recapitulate the immune constituents of human GBM.

### Comparison of the diversity of immune species in QPP and human disease.

To quantify the diversity of immune species in our cohorts, we calculated the Shannon Diversity Index (SDI) for each data set (Supplemental Table 10). The greatest diversity in immune cell populations was identified in the GBM cohort (SDI = 10.2779). The immune compartment was less diverse in QPP7 allograft tumors (SDI = 8.54) and in spontaneous QPP tumors (SDI = 7.16). When combined, the diversity of the combined QPP7 allograft and spontaneous QPP data set was similar to that of the QPP7 allograft tumors alone (SDI = 8.76). Analysis using the χ^2^ test determined that the heterogeneity was statistically significantly different among the 3 data sets (Supplemental Table 10).

To identify high-level signaling pathways that are conserved between our combined murine models and human GBM, we performed Gene Ontology (GO) analysis of the upregulated genes from each subtype cluster (30) (Supplemental Tables 11 and 12). GO analysis identified that canonical immune function pathways, including immune response (cluster 0, mouse; cluster 8, human), innate immune response (cluster 0, mouse; cluster 8, human), and response to external stimulus (cluster 3, mouse; cluster 9, human), were consistently upregulated in myeloid cells. Finally, we also observed potentially novel GO pathways, such as peptide signaling, in both myeloid and lymphoid arms in both species. Overall, our findings show that the immune compartments of tumors derived from spontaneous and QPP7 tumors are similar to those found human GBM.

We have prepared a table comparing the 3 systems frequently used to study GBM (Table 1). Specifically, we reference the origin, TMB, and immune infiltrate.

## Discussion

In this study, we characterized the immune compartment of spontaneous and allograft QPP tumors alongside human GBM samples to determine whether QPP tumors represent an improved preclinical tool to model and study the immunogenic diversity of human GBM. The limited availability of animal models that faithfully recapitulate the pathogenesis, histopathology, and development of GBM likely contribute to the poor clinical success rate of candidate drugs against this deadly disease. To address this resource gap, we previously developed the QPP GEMM, which spontaneously develops heterogeneous tumors with features similar to human GBM (9). We encourage others to characterize other available models, as there is a paucity of models in general for gliomas and, furthermore, for studies that require an intact immune system.

First, we created the QPP7 cell line model, which we derived from a spontaneous tumor from a QPP GEMM. We used QPP7 to derive secondary tumors by implanting cells into the striatum of C57BL/6J. IHC and scSEQ analyses of both spontaneous QPP and allograft QPP7 tumors showed that, for both models, the immune compartment closely approximated that found in human GBM. Specifically, both mouse tumor models and human GBM tissue were characterized by an immune compartment dominated by immunosuppressive myeloid cells, with relatively smaller populations of NK, B, and T cells. This finding is consistent with other recent studies of human GBM (31, 32). Subtype analysis of the myeloid compartment demonstrated similar complexity and proportions of myeloid cell subtypes between the mouse and human glioma cohorts.

Interestingly, although the immune cell populations were similar between mouse and human tumors, they appear to be driven by different transcriptomic programs (i.e., CSFR3^+^ neutrophils in humans compared with Cxcl12^hi^Cd14^hi^ neutrophils in mice). We observed an enrichment of neutrophils, T cells, and NK cells in QPP7 allograft tumors compared with spontaneous QPP tumors. In human tumor tissue, we observed increased lymphoid and neutrophil infiltration in HGG (grade 3 or 4) tumors compared with LGG tumors as well as a shift in the myeloid population from one of a more microglia-dominant signature to a more macrophage-dominant signature (33). This may indicate that the QPP7 allograft model, which has been cultured ex vivo, has increased aggressiveness compared with the spontaneous QPP model.

Slight variations were observed among the 3 tumor types. Higher neutrophil infiltration was detected in QPP-derived tumors compared with human tumors. This discrepancy might reveal a bona fide overrepresentation of intratumoral neutrophils in the QPP model, and/or it could indicate a methodological artifact. For example, it could result from the high overlap of MDSC and neutrophil transcriptomic profiles. Differences in the immune constituents between spontaneous and allograft QPP tumors might also result from the microenvironmental response to the mechanical implantation procedure or from the different tumor progression trajectories of these tumors. The spontaneous model presumably initiates from a few cells transformed by cre-lox recombination and then progresses into an aggressive tumor, whereas the implanted tumor starts with a bolus of 50,000 cells that have survived the selective pressure of serial passaging in vitro. Of note, implanted QPP tumors were enriched for neutrophils, T cells, and NK cells compared with spontaneous QPP tumors. This increase in lymphoid and neutrophil infiltration, as well as a shift from a microglia-dominant to a more macrophage-dominant myeloid population, was also observed when we compared HGG tumors with LGG tumors. Overall, these findings and the survival curve shortening suggest that implanted QPP7 cells result in a more aggressive disease compared with the spontaneous QPP model, and, thus, QPP7 allograft tumors may be more suitable for studying aggressive GBM.

GO analysis of QPP-derived and patient GBM samples showed enrichment in gene clusters with canonical immune functions, such as immune response, defense response, and regulation of immune system processes, in all 3 cohorts. Surprisingly, our GO analysis also found upregulation of peptide synthesis in the T cell compartment as well as peptide binding in the myeloid compartment in all 3 cohorts, suggesting a signaling mechanism is active that warrants further investigation.

Tumors derived from our spontaneous and QPP7 allograft models better recapitulate human disease compared with available GBM models. Currently, the most prevalent GBM model is the GL261 mouse glioma system. The GL261 cell line was generated by injecting methylcholanthrene into the brains of C57BL/6J mice to induce tumors, and divergent clones subsequently derived from the resulting tumor have been used by various groups (34). GL261 tumors harbor activating *Kras* mutations (expressed in ~1% of patients) and have a high TMB (GL261, 4978/Mb; GBM, 2.7/Mb). They express high MHC-1 protein levels, but GL261 tumors have relatively limited expression of other immune antigens, including MHC II, B7-1, and B7-2, resulting in a limited, immunogenic phenotype. These characteristics of GL261 tumors, which vary in important ways from most GBM tumors, could explain why GL261 mouse models respond to immunotherapeutics that have been shown to be ineffective for patients with GBM. As previously described, our QPP GEMM animals develop heterogeneous tumors, with features like all 4 human GBM subtypes recapitulated in different mice. It is thus reasonable to speculate that further characterization of our QPP model, as well as of human GBM tumors, may further support that the QPP model offers an opportunity to model immunogenically diverse populations, such as those represented in human disease, as shown by our IHC and scSEQ findings for the cohorts in this study (9).

Other spontaneous models of GBM include the sleeping beauty transposase-mediated model (35) and virally induced systems, such as AAV or RCAS (36). The latter systems typically rely on strong mutagenic drivers that are not common in human GBM, such as mutant *Kras*. By contrast, our spontaneously arising QPP model is induced by deletion of 3 key tumor-suppressor genes that are frequently mutated/deleted in patients with GBM (1, 10). Further, our QPP-derived tumors harbor a TMB more similar to human disease compared with GL261 tumors, and our findings here show that the intratumoral immune constituents of QPP-derived tumors are similar to those found in human GBM.

Recent back-to-back publications (31, 32) showed that patients with GBM have a highly variable immune infiltrate composed of myeloid, neutrophil, and T cell components, with smaller proportions of other immune populations present. Friebel et al. performed CyTOF analysis to produce a protein-level view of the immune cell populations in primary and metastatic lesions and in *IDH* wild-type versus *IDH* mutant tumors (31). While Klemm et al. investigated the transcriptomic changes that occur with different tumor origin and *IDH* status, the QPP models are *Idh* wild type (32). These observations are in line with what was observed in our data set.

This study validates the QPP model as an important preclinical tool to study the immune microenvironment of GBM. Like human GBM tumors, QPP tumors are characterized by a predominantly myeloid-infiltrating population with smaller T, B, and NK cell populations. On the basis of their similarity to human tumors, spontaneous QPP and QPP7 allograft models could improve the accuracy of preclinical studies to predict clinical outcomes for therapeutics and, particularly, for immunotherapeutic strategies for patients with GBM.

## Methods

### Materials availability

#### Cell line.

The QPP7 cell line was generated in-house in 2017 by culturing tumor cells from QPP mice in NeuroCult Basal Medium (mouse and rat; catalog 05700; Stemcell Technologies Inc.) with NeuroCult Proliferation Supplement (mouse and rat; Stemcell Technologies Inc.) added. Cells were cultured as spheres and subcultured every 2–3 days at a 1:5 ratio with Accutase (Innovative Cell Technologies Inc.) as the dissociation solution. As the cells are primary, they have not been banked, tested, or authenticated.

### Data and code availability

#### Data set.

The scSeq data set has been deposited in the GEO database (GSE147275).

#### Code.

Code used to generate figures and analyze data is available at https://github.com/zamlerd/Single_Cell_Sequencing (commit ID 6dbce63).

### Experimental model and patient details

#### Mouse models.

The QPP spontaneous glioma model exists on a mixed background and is maintained in-house at the MD Anderson Cancer Center in the Department of Cancer Biology. Frozen sperm have also been deposited in the MD Anderson Mouse Transgenics Core.

#### Patient data.

Patient information, including sex, genomic information, and site of resection, etc., is available in Supplemental Table 1.

### Key resources

Details regarding antibodies are provided in Supplemental Table 2. Biological samples, including patient tissue, were provided by the MD Anderson Cancer Center. Critical commercial assays included the Chromium Single Cell 3′ Reagent Kit (10x Genomics) and the ImmPACT NovaRED HRP substrate (Vector Laboratories). C57BL/6J mice were obtained from The Jackson Laboratory (strain 000664).

### Software and algorithms

#### Rstudio.

We used the Rstudio software package, which can be obtained from https://www.rstudio.com/

#### Cell ranger.

We used the Cellranger software package, which can be obtained from https://support.10xgenomics.com/single-cell-gene-expression/software/overview/welcome

#### Seurat.

We used the Seurat software package, which can be obtained from https://satijalab.org/

#### Custom pipeline.

We also developed a custom pipeline which can be found at https://github.com/zamlerd/Single_Cell_Sequencing/blob/master/Aggregate_All_JCII.R (commit ID 6dbce63).

### Murine glioma models

To trigger the spontaneous QPP gliomas, tamoxifen was dissolved in corn oil at a concentration of 10 mg/mL and injected subcutaneously in a total volume of 20 μL in P7 and P8 mice to induce glioma development in the Nes-CreERT2; Qk^L/L^; Pten^L/L^; Trp53^L/L^ background. The mice were monitored for neurological symptoms or other signs of ill health every other day and were euthanized and necropsied when moribund. To implant QPP gliomas, cells were cultured as described above until the time of surgical implantation. The QPP cells were dissociated with Accutase (Innovative Cell Technologies Inc.) for 5 to 10 minutes at room temperature, the Accutase was neutralized by dilution with medium, and the cells were pelleted by centrifugation. Automated cell counting was performed, and the cells were resuspended at a concentration of 2.5 × 10^4^ cells per μL. Mice were anesthetized with a combination of ketamine and xylazine, and 5 × 10^4^ QPP cells were implanted at the stereotactic coordinates of +0.5 mm forward and +2 mm lateral right from the bregma at a depth of 3 mm. After the anesthesia was reversed with atipamezole, the mice were monitored until signs of tumor burden appeared, at which point they were euthanized by transcardial perfusion with Tyrode’s solution (MilliporeSigma; Tyrode’s solution is a salt formulation designed to keep the heart beating while flushing blood from the mouse’s circulatory system during transcardial perfusion). Mouse brains were removed and fixed in paraformaldehyde.

### IHC

Tissues were embedded in paraffin, serially sectioned on a microtome at 5 μM, and stained with H&E. Specifically, sections on microscope slides were stained with freshly filtered hematoxylin for 30 seconds and then with eosin for 15 seconds before dehydration in two 1-minute washes in 95% ethanol, followed by three 1-minute washes in 100% ethanol and, finally, 3 quick rinses in xylene before application of cover slips to slides. For antibody staining, tissue sections were baked for 1 hour at 60°C before being washed 3 times in xylene for 5 minutes, followed by washes in 100%, 95%, 70%, and 50% ethanol and then tap water. Antigen retrieval was performed using a Biogenix easy-retrieval microwave set at 95°C for 10 minutes in sodium citrate buffer (Poly Scientific) at pH 6.0. Slides were then washed in PBS for 5 minutes before being blocked with 3% BSA at room temperature for 1 hour. Slides were then incubated overnight at 4°C at the dilutions listed in Supplemental Table 2. The next morning, slides were washed 3 times in PBS with 0.1% Tween for 5 minutes before incubation with the appropriate HRP-conjugated secondary antibody for 1 hour at room temperature. Slides were then washed 3 times in PBS for 5 minutes before being developed using the NovaRED chromagen incorporation kit (Vector Laboratories). Cover slips were then applied to the slides in aqueous mounting medium. For our IHC analysis, we quantified the number of cells by hand in a 396 mm^2^ area (×20 field). Antibody catalog numbers, dilutions, and RRIDs (RRID denotes a number used by the Resource Identification Portal) can be found in Supplemental Table 2.

### scSeq

Mice with implanted QPP tumors were perfused with Tyrode’s solution before the tumors were reduced to a single-cell suspension and frozen in Bambanker cell-freezing medium (Wako Chemicals USA Inc.) at -80°C. Gliomas were mechanically dissociated with scissors while suspended in Accutase solution (Innovative Cell Technologies Inc.) at room temperature and then serially drawn through 25, 10, and 5 mL pipettes before being drawn through an 181/2-gauge syringe. After 10 minutes of dissociation, cells were spun down at 420*g* for 5 minutes at 4°C and then resuspended in 10 mL of a 0.9 N sucrose solution and spun down again at 800*g* for 8 minutes at 4°C with the brake off. Once a sufficient number of samples was accumulated to be run in the 10x pipeline (10x Genomics), cells were then thawed and resuspended in 1 mL PBS containing 1% BSA for manual counting. Cells were then stained with CD45 antibody (BD Biosciences, catalog 555482) at 1:5 for human cells (Tonbo Biosciences, catalog 50-0454-U100) or at 1:10 for mouse cells for 20 minutes on ice. Samples had Sytox blue added just before sorting so that only live CD45^+^ cells would be collected. Cells were then sorted in a solution of 50% FBS and 0.5% BSA in PBS, spun down, and resuspended at a concentration of 700 to 1200 cells/μL for microfluidics on the 10x platform (10x Genomics). The 10x protocol, which is publicly available (https://assets.ctfassets.net/an68im79xiti/2NaoOhmA0jot0ggwcyEKaC/fc58451fd97d9cbe012c0abbb097cc38/CG000204_ChromiumNextGEMSingleCell3_v3.1_Rev_C.pdf), was followed to generate the cDNA libraries that were sequenced.

The libraries were sequenced on an Illumina NextSeq 500, and up to 4 indexed samples were multiplexed into 1 output flow cell using the Illumina high-output sequencing kit (V2.5) in paired-end sequencing (R1, 26nt; R2, 98nt; and i7 index, 8nt) per the manufacturer’s instructions (3′ Single-cell RNA sequencing kit, 10x Genomics).

The data were then analyzed using the cellranger pipeline (10x Genomics) to generate gene count matrices. The mkfastq argument (10x Genomics) was used to separate individual samples with simple csv sample sheets to indicate the well that was used on the i7 index plate to label each sample. The count argument (10x Genomics) was then used with the expected number of cells for each mouse or patient. For the mice, the expected cell numbers were 10,000, whereas for patients, the numbers varied between 2000 and 8000. Mice were aligned with the mm10 genome, and humans were aligned with GRCh38. The aggr argument (10x Genomics) was then used to aggregate samples from each condition (spontaneous QPP, implanted QPP, and patient) for further analysis. Once gene count matrices were generated, they were read into an adapted version of the Seurat pipeline (37, 38) for filtering, normalization, and plotting. Genes that were expressed in fewer than 3 cells were ignored, and cells that expressed fewer than 200 genes or more than 2500 genes were excluded, to remove potentially poor- and high-PCR artifact cells, respectively. Finally, to generate a percentage of mitochondrial DNA variability and to exclude any cells with more than 25% mitochondrial DNA (as these may be doublets or low-quality dying cells), cells were normalized using regression to remove the percentage of mitochondrial DNA variable via the scTransform (39) command. Next, the cell clusters were identified and visualized using SNN and UMAP, respectively, before generating a list of differentially expressed genes for each cluster. A list of differentially expressed genes was generated to label our clusters at a low resolution of 0.1. These clusters’ labels were based on at least 3 differentially expressed genes, and violin plots were generated to show the relative specificity to the cluster.

Identification of the clusters was as follows: neutrophils (CD24a, S100a8, and S100a9); APCs (CD74 [MHCII], H2-Eb1, and H2-Aa); T cells (Cd3d, Cd3e, and Cd3g); and microglia and macrophages (Cd68, Cx3cr1, and Tmem119). For analyses performed on the combination of implanted and spontaneous QPP models, we joined the data sets (using the FindIntegrationAnchors command to determine genes that can be used to integrate 2 data sets — after the determination of the anchors used the IntegrateData command) with the aforementioned anchors to combine our 2 data sets. Data were then normalized using the scTransform command, which uses regression analysis to scale the expression of all genes based on linear regression to mitochondrial RNA expression. Data sets were then processed for principal component analysis (PCA) with the RunPCA command, and elbow plots were printed with the Elbow plot command in order to determine the optimal number with principal components for clustering. Data sets then were submitted to cluster analysis with RunUMAP and FindNeighbors commands before FindClusters was run with either 0.1 or 0.65 resolution for low- and high-resolution clustering, respectively. Differentially expressed genes were identified using cutoffs for min.pct = 0.25 and logfc.threshold = 0.25. Plots were generated with the DimPlot, FeaturePlot, or VlnPlot commands. For consistency, the same markers (or homologs) were used to designate these populations regardless of the species, with the following exceptions: CD24a and ITGAX were used interchangeably to label neutrophil clusters, and Nktr and HCST were used to label NK clusters in mice and humans, respectively.

### Gene ontology analysis

Gene ontology analyses were performed as previously described, and publicly available code was adapted to our data set (32).

### Statistics

Statistics were performed using the χ^2^ test and an unpaired 2-tailed *t* test with Welch’s correction. *P* values of less than 0.05 were considered significant.

### Approval

This study was performed with Institutional Animal Care and Use Committee approval from The University of Texas MD Anderson Cancer.

## Author contributions

DBZ, CK, MO, KT, JL, YL, IL, CY, KR, ABH, GFD, JH, TS, LMK, KH, RZV, and BYSK provided experimental design and/or implementation. DBZ, JH, ABH, GFD, SSW, and LMK analyzed and interpreted the data. DBZ, ABH, GFD, and JH wrote the manuscript.

## Supplementary Material

Supplemental data

Supplemental tables 1-12

## Figures and Tables

**Table 1:**
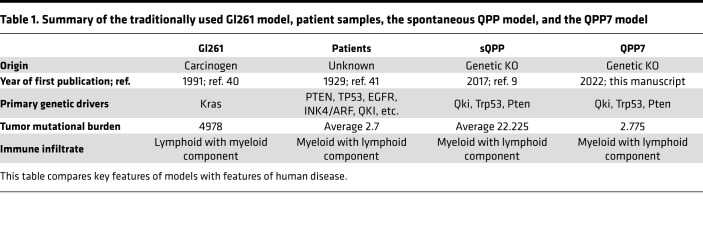
Summary of the traditionally used Gl261 model, patient samples, the spontaneous QPP model, and the QPP7 model

## Version 1 06/02/2022

In-Press Preview

## Version 2 06/22/2022

Electronic publication
