# Supplementary material for: Immune landscape of a genetically engineered murine model of glioma compared with human glioma
Source: JCI Insight. 2022 Jun 22;7(12):e148990. doi: 10.1172/jci.insight.148990 (PMC9309065; doi:10.1172/jci.insight.148990)
Supplement: Supplemental data [file jciinsight-7-148990-s104.pdf]

Supplementary Figure 1: Histopathological staining of representative QPP gliomas arising spontaneously or implanted in mice, displaying the hallmark features of glioblastoma. a. Representative hematoxylin and eosin (H & E)-stained whole-mount coronal section of brain with spontaneous QPP tumor at 1x magnification. **b.** Necrosis of the spontaneous tumor. **c.** Invasive infiltrating edge of the spontaneous tumor marked by green arrows. **d.** Ki67 staining within the spontaneous QPP glioma demonstrating the high number of active proliferating cells. **e.** Representative H & E-stained whole-mount coronal section of brain with implanted QPP tumor at 1x magnification **f.** Necrosis of the QPP implanted tumor. **g.**

Invasive infiltrating edge of the implanted QPP tumor. **h.** KI67 staining within the implanted QPP border to determine location of proliferating cells. (scale bar = 200 µM).

Supplementary Figure 2: Survival and tumor mutational burden (TMB) of QPP Models. **a.** Survival of n=15 QPP mixed background mice injected with 20uL subcutaneous tamoxifen on P7 and P8. Survival of n=9 C57Bl6/j mice implanted with 50,000 QPP7 cells into their striatum. **b.** Tumor mutational burden of n=5 Spontaneous QPP mice and the QPP7 cell line.

Supplementary Figure 3: Immune profiling of spontaneous and implanted QPP tumors and human glioblastoma. a-c. Representative images of immunohistochemistry (IHC) analysis of tumors from (a) spontaneous and (b) implanted QPP models as well as from glioma patients**.**

Supplementary Figure 4: Comparison of freshly isolated naïve mouse brains and previously frozen naïve mouse brains. a. UMAP shows aggregated clustering of CD45+ cells of n=2 freshly isolated naïve mouse brains and n=2 previously frozen mouse brains. **b.** Demonstrates the contributions of individual mice to **a**.

Supplementary Figure 5: Quality control metrics for the spontaneous QPP dataset. a. Featureplot showing the counts of RNA molecules detected per cell. **b.** Featureplot showing the number of unique genes expressed in each cell. **c.** Featureplot showing the percent of heat shock proteins expressed in each cell. **d.** Featureplot showing the percent of mitochondrial RNA molecules for each cell. **e.** Barplot showing the number of cells for each cluster for individual mice. **f.** Violin plot showing the distribution of (left) unique gene counts, (center) RNA molecule counts, and (right) mitochondrial RNA percentage for the dataset. **g.** Scatter plots showing the

correlation of (left) mitochondrial percentage to total RNA counts and (right) unique genes to total RNA counts after normalization.

Supplementary Figure 6: Immune constituents of spontaneous QPP tumors. a. UMAPS showing aggregate of CD45+ immune infiltrates from n=3 spontaneous QPP tumors at moribund timepoint. **b.-g.** UMAPS show (b) microglia and macrophage clusters identified by Cd68, Cx3cr1, and Tmem119 markers; (c) neutrophil clusters identified by Cd24a, S100a8, and S100a9 markers; (d) APC clusters identified by Cd74, H2-Eb1, and H2-Aa markers; (e) T-cell clusters identified by Cd3d, Cd3e, and Cd3g markers; (f) natural killer cell clusters identified by Klrd1, Nkg7, and Nktr markers; and (g) UMAPS show B-cell clusters identified by Cd79a, Cd79b, and Ms4a7 markers. All violin plots show the specificity of given markers for macrophages (red), microglia (green), APC (blue), and neutrophils (purple).

Supplementary Figure 7: Quality control metrics for the implanted QPP dataset. a. Featureplot showing the counts of RNA molecules detected per cell. **b.** Featureplot showing the number of unique genes expressed in each cell. **c.** Featureplot showing the percent of heat shock proteins expressed in each cell. **d.** Featureplot showing the percent of mitochondrial RNA molecules for each cell. **e.** Barplot showing the number of cells per cluster for individual mice. **f.** Violin plot showing the distribution of (left) unique gene counts, (center) RNA molecule counts, and (right) mitochondrial RNA percentage for the dataset. **g.** Scatter plot showing the correlation of (right) mitochondrial percentage to total RNA counts and (left) unique genes to total RNA counts after normalization.

Supplementary Figure 8: Immune constituents of implanted QPP tumors. a. UMAPs showing aggregate of CD45+ immune infiltrates from n=3 Implanted QPP tumors at moribund

timepoint. **b-h.** UMAPS show (b) microglia and macrophage clusters identified by Cd68, Cx3cr1, and Tmem119 markers; (c) neutrophil clusters identified by Cd24a, S100a8, and S100a9 markers; (d) antigen-presenting cell clusters identified by Cd74, H2-Eb1, and H2-Aa markers; (e) lytic myeloid clusters identified by Ly6a, Ly6c2, and Ly6d markers; (f) natural killer (NK) cell clusters identified by Klrd1, Nkg7, and Nktr markers; (g) B-cell clusters identified by Cd79a, Cd79b, and Ms4a7 markers; and (h) T cell clusters identified by Cd3d, Cd3e, and Cd3g markers. All violin plots show the specificity of given markers for neutrophils (red), macrophages/microglia (lime), T and NK cells (green), APCs (blue), and lytic myeloid cells (purple).

Supplementary Figure 9: QPP7 Tumors Show Macroscopic Heterogeneity. a-b. QPP7 tumors harvested at the moribund endpoint of a mouse implanted with QPP7 cells. The tumor in panel a has much more hemorrhaging and necrosis compared to that in (b).

Supplementary Figure 10: Quality control metrics for the combined QPP dataset. a. Featureplot showing the counts of RNA molecules detected per cell. **b.** Featureplot showing the number of unique genes expressed in each cell. **c.** Featureplot showing the percent of heat shock proteins expressed in each cell. **d.** Featureplot showing the percent of mitochondrial RNA molecules for each cell. **e.** Barplot showing the number of cells per cluster for individual mice.

Supplementary Figure 11: Comparison of QPP Immune Constituents. a. UMAPs show aggregate of CD45+ immune infiltrates from n=3 spontaneous QPP tumors and n=3 Implanted QPP tumors at moribund timepoint. **b-h.** UMAPS show (b) neutrophil clusters identified by Cd24a, S100a8, and S100a9 markers; (c) microglia and macrophage clusters identified by Cd68, Cx3cr1, and Tmem119 markers; (d) antigen-presenting cell clusters identified by Cd74,

H2-Eb1, and H2-Aa markers; (e) lytic myeloid clusters identified by Ly6a, Ly6c2, and Ly6d markers; (f) natural killer (NK) cell clusters identified by Klrd1, Nkg7, and Nktr markers; (g) B-cell clusters identified by Cd79a, Cd79b, and Ms4a7 markers; and (h) T cell clusters identified by Cd3d, Cd3e, and Cd3g markers. All violin plots show the specificity of given markers for neutrophils (red), macrophages/microglia (lime), T and NK cells (green), APCs (blue), and lytic myeloid cells (purple).

Supplementary Figure 12: Heterogeneity between QPP mice and glioblastoma (GBM) patients. **a.** Demonstrates the contribution of the individual spontaneous QPP mice to the aggregate plot in **Figure 3a**. **b.** Demonstrates the contribution of the individual implanted QPP mice to the aggregate plot in **Figure 3b**. **c.** Demonstrates the contribution of the individual GBM patients to the aggregate plot in **Figure 3d**.

Supplementary Figure 13: Quality control metrics for the glioblastoma (GBM) patient dataset. a. Featureplot showing the counts of RNA molecules detected per cell. **b-h.** UMAPs show (b) T cell clusters identified by CD3D, CD3E, and CD3G markers, (c) neutrophil clusters identified by ITGAX, S100A8, and S100A9 markers; (d) natural killer (NK) cell clusters identified by HCST, KLRD1, and NKG7 markers; (e) antigen-presenting cell clusters identified by CD74, HLA-DRA, and HLA-DRB1 markers; (f) microglia clusters identified by CD14, MRC1, and MARCKS markers; (g) macrophage clusters identified by CD68, CX3CR1, and TMEM119 markers; and (h) B-cell clusters identified by CD79A, CD79B, and MS4A1 markers. All violin plots show the specificity of color-coded given markers.

Supplementary Figure 14: Immune constituents of human glioma. a. UMAPs show aggregate of CD45+ immune infiltrates from n=15 glioma patients at surgical resection. **b-h.**

UMAPS show (b) T cell clusters identified by CD3D, CD3E, and CD3G markers, (c) neutrophil clusters identified by ITGAX, S100A8, and S100A9 markers; (d) natural killer (NK) cell clusters identified by HCST, KLRD1, and NKG7 markers; (e) antigen-presenting cell clusters identified by CD74, HLA-DRA, and HLA-DRB1 markers; (f) microglia clusters identified by CD14, MRC1, and MARCKS markers; (g) macrophage clusters identified by CD68, CX3CR1, and TMEM119 markers; and (h) B-cell clusters identified by CD79A, CD79B, and MS4A1 markers. All violin plots show the specificity of color-coded given markers.

Supplementary Figure 15: Comparison of human GBM and human low-grade glioma (LGG) immune constituents. a. UMAPs show aggregate of CD45+ immune infiltrates from n=9 GBM patients at surgical resection compared to n=6 LGG patients at the same time point. **b.** UMAPs show the immune constituents GBM and LGG patients are overlaid to better display the similarities and differences between the groups. **c.** UMAPs identifying lymphoid clusters in samples from GBM (high-grade glioma; HGG) and LGG patients. **d.** UMAPs identifying myeloid clusters in samples from GBM (high-grade glioma; HGG) and LGG patients. **e.** Bar plot of the number of cells per cluster in samples from GBM (HGG) or LGG patients.

Table 1: Comparison of Glioma Models to Patients. For ease of comparison please find several of the key features of the models discussed to the patient disease.

Funding: This research was supported by the National Institutes of Health (CA1208113, P30 CA016672, R37CA214800, CA18689204) the Ben and Catherine Ivy Foundation, The University of Texas MD Anderson Cancer Center GBM Moonshot, the Sontag Foundation, the Brockman Foundation.

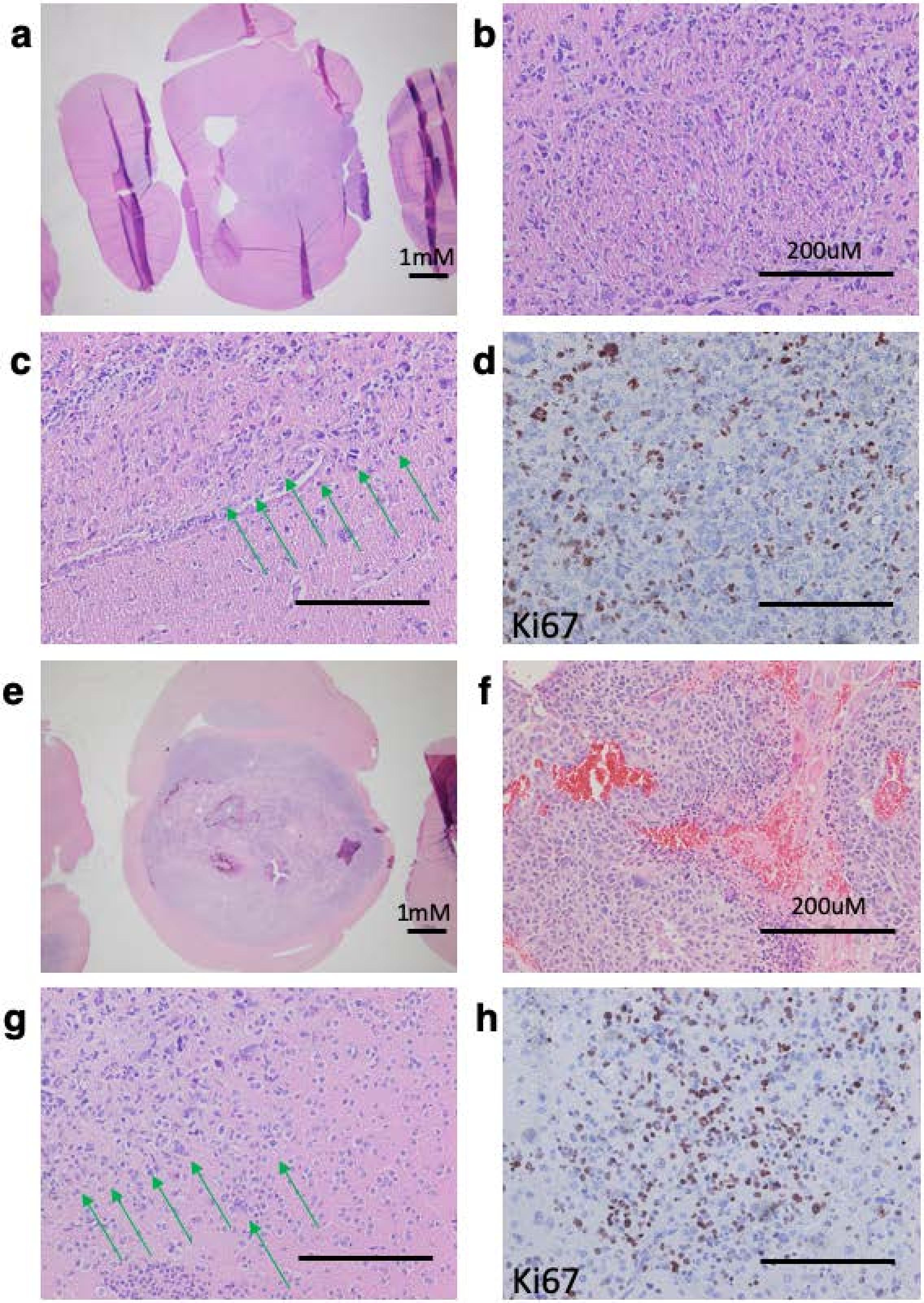

Supplementary Figure 1

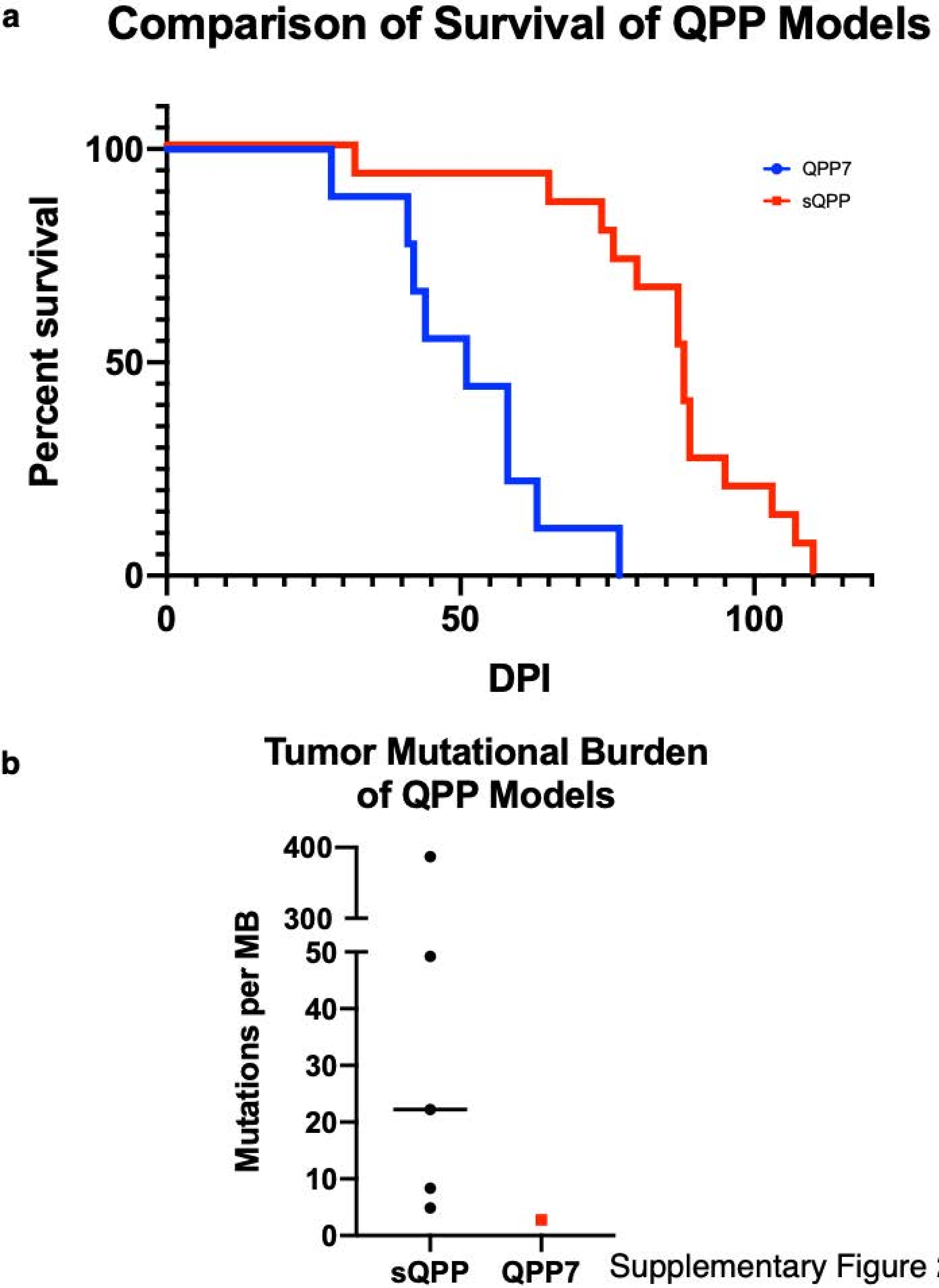

Supplementary Figure 2

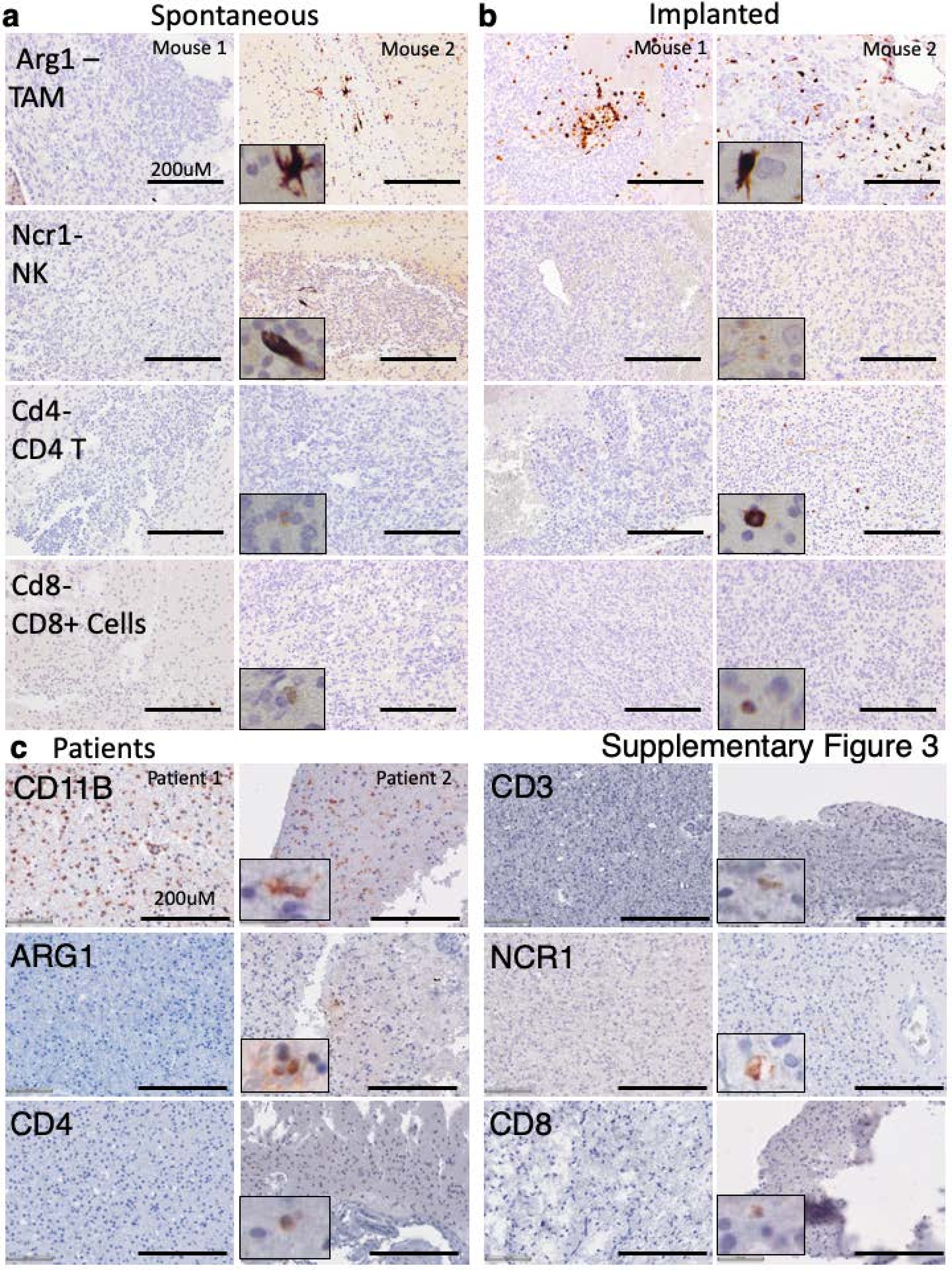

Supplementary Figure 3

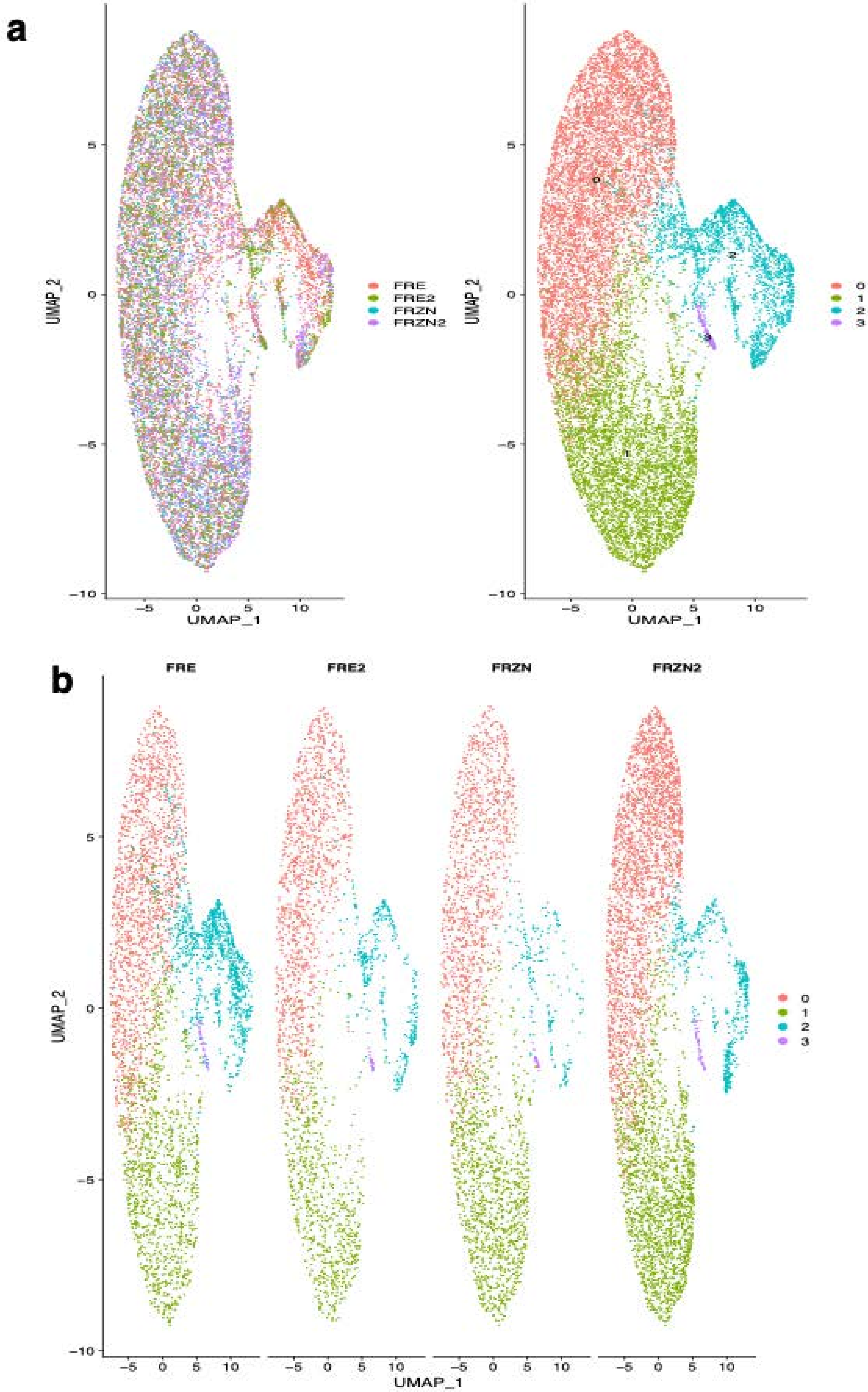

Supplementary Figure 4

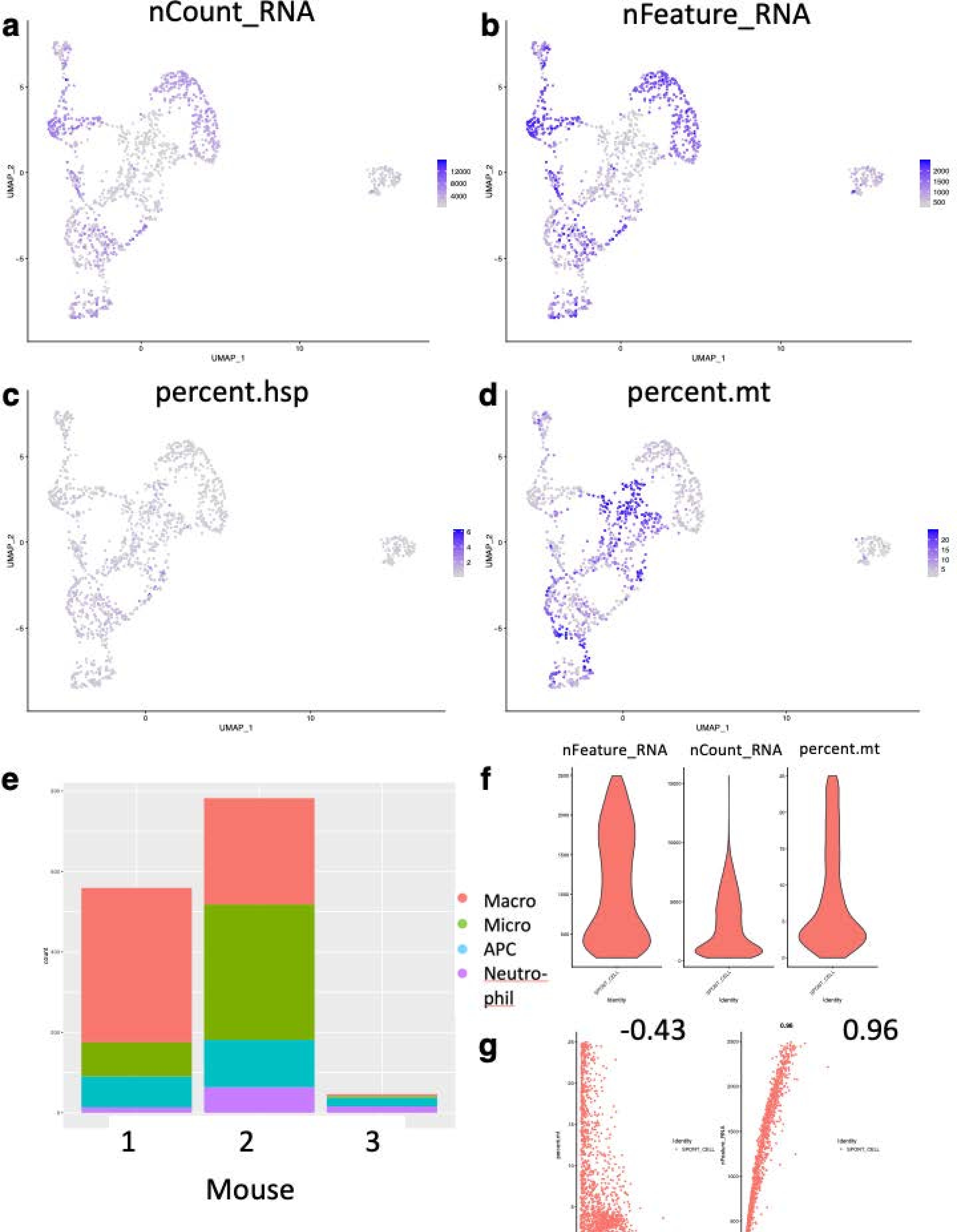

Supplementary Figure 5

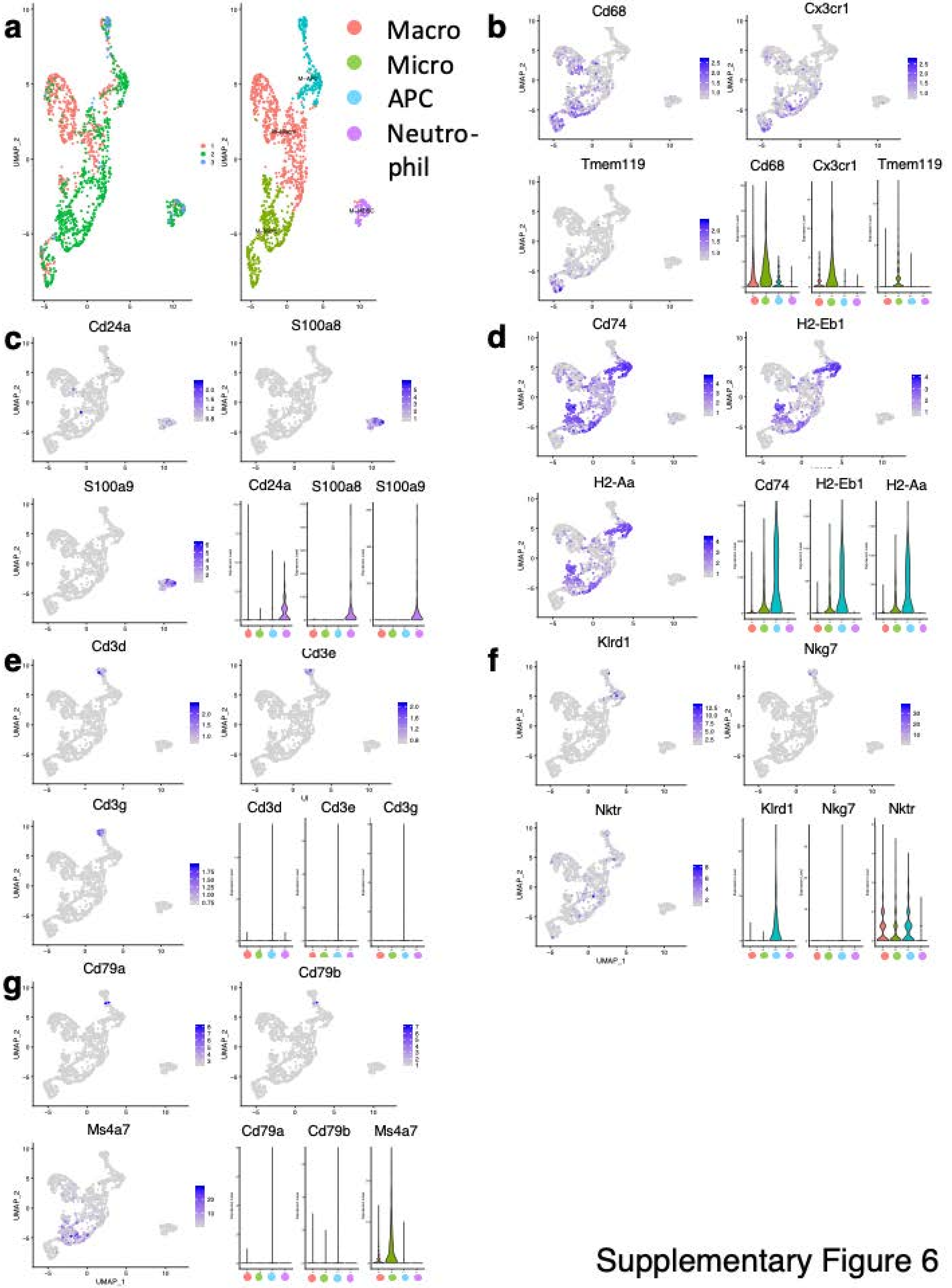

Supplementary Figure 6

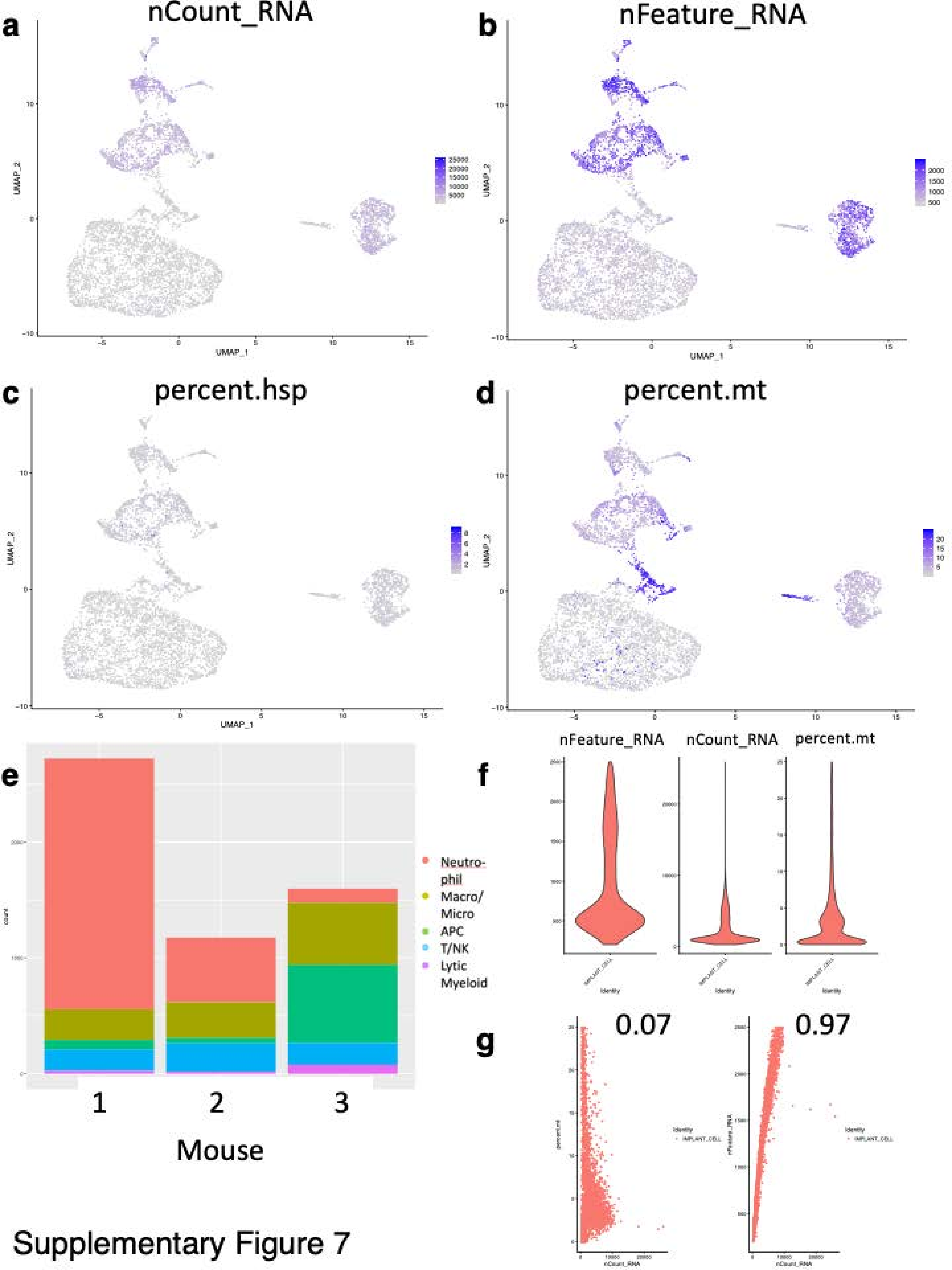

Supplementary Figure 7

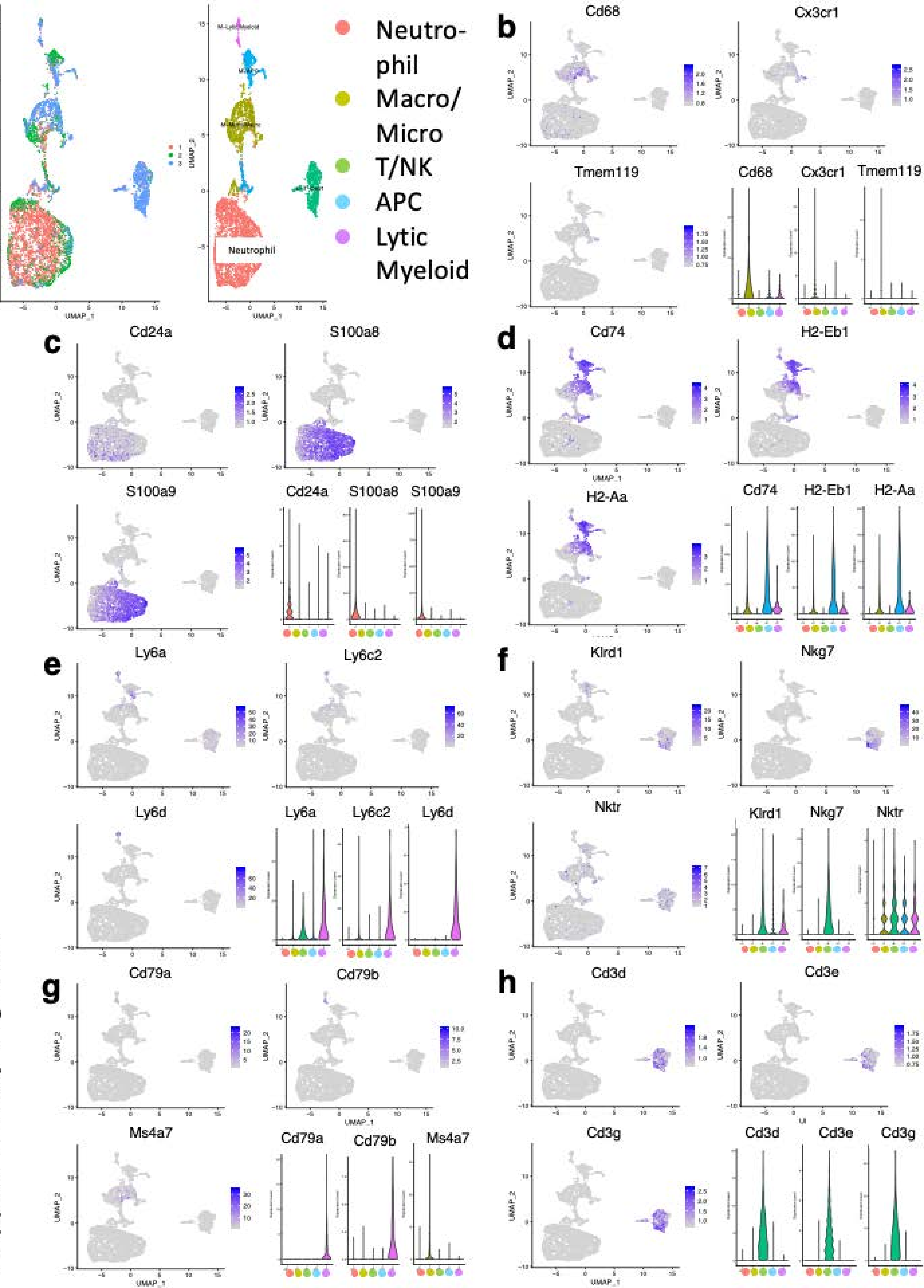

Supplementary Figure 8

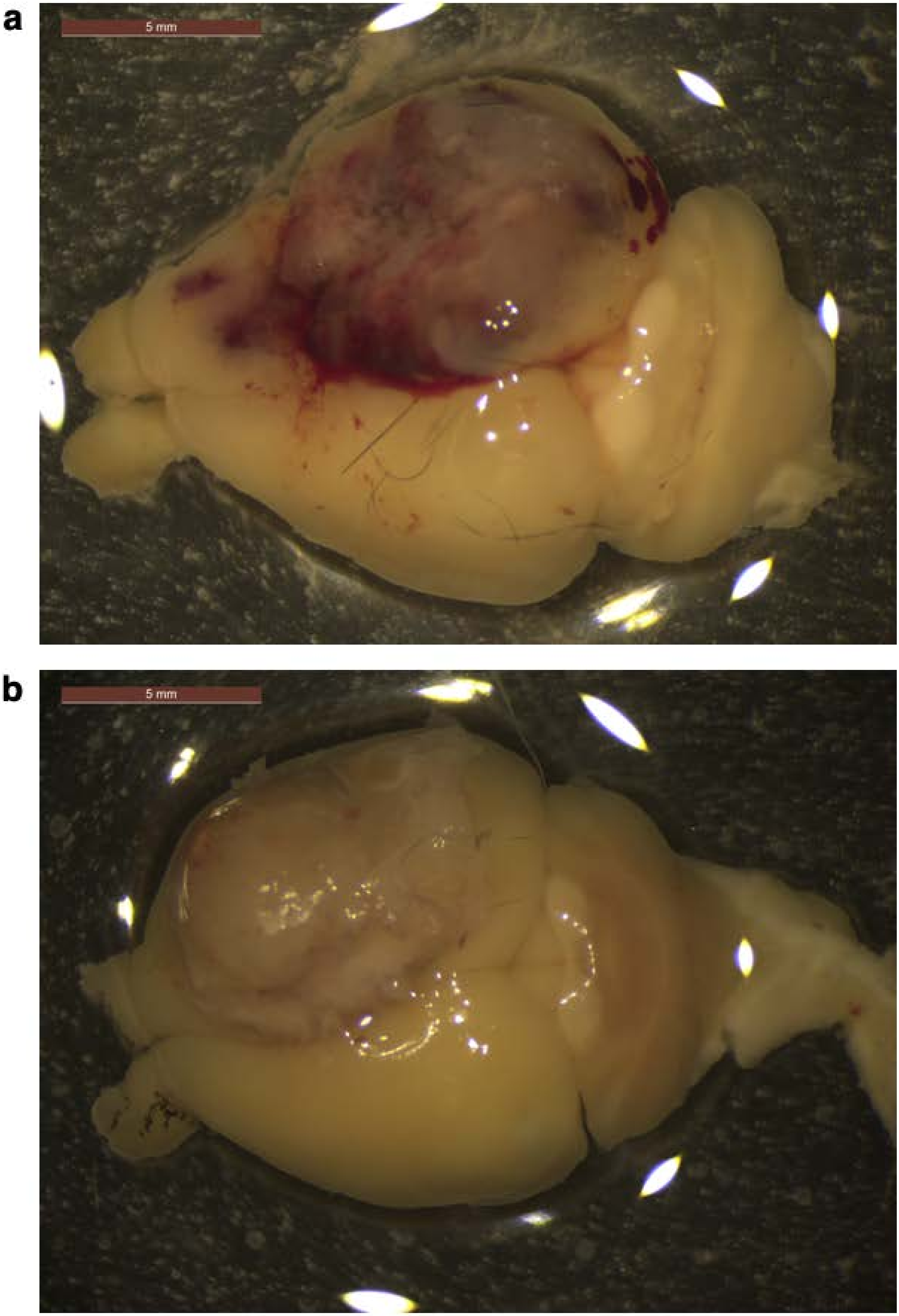

Supplementary Figure 9

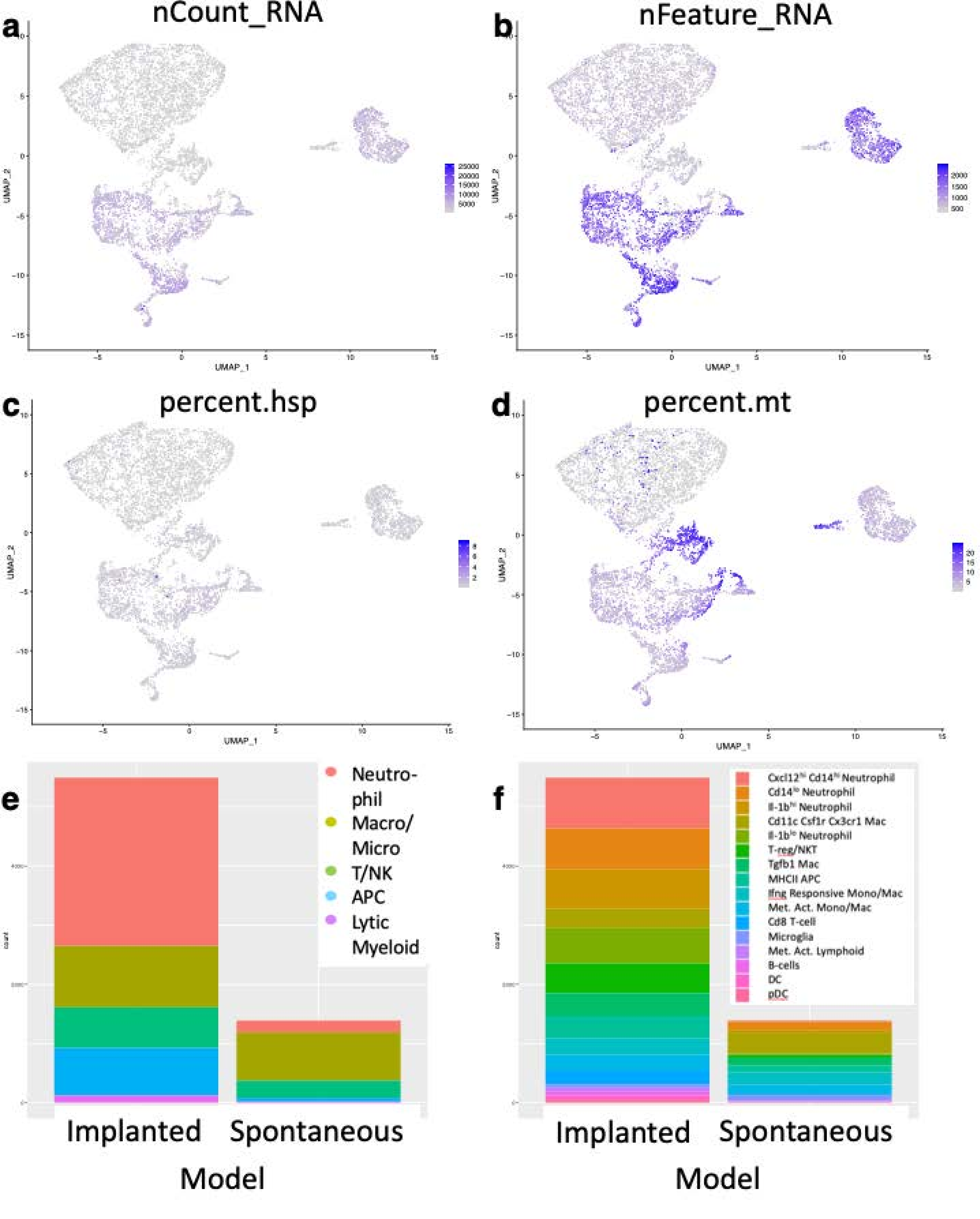

Supplementary Figure 10

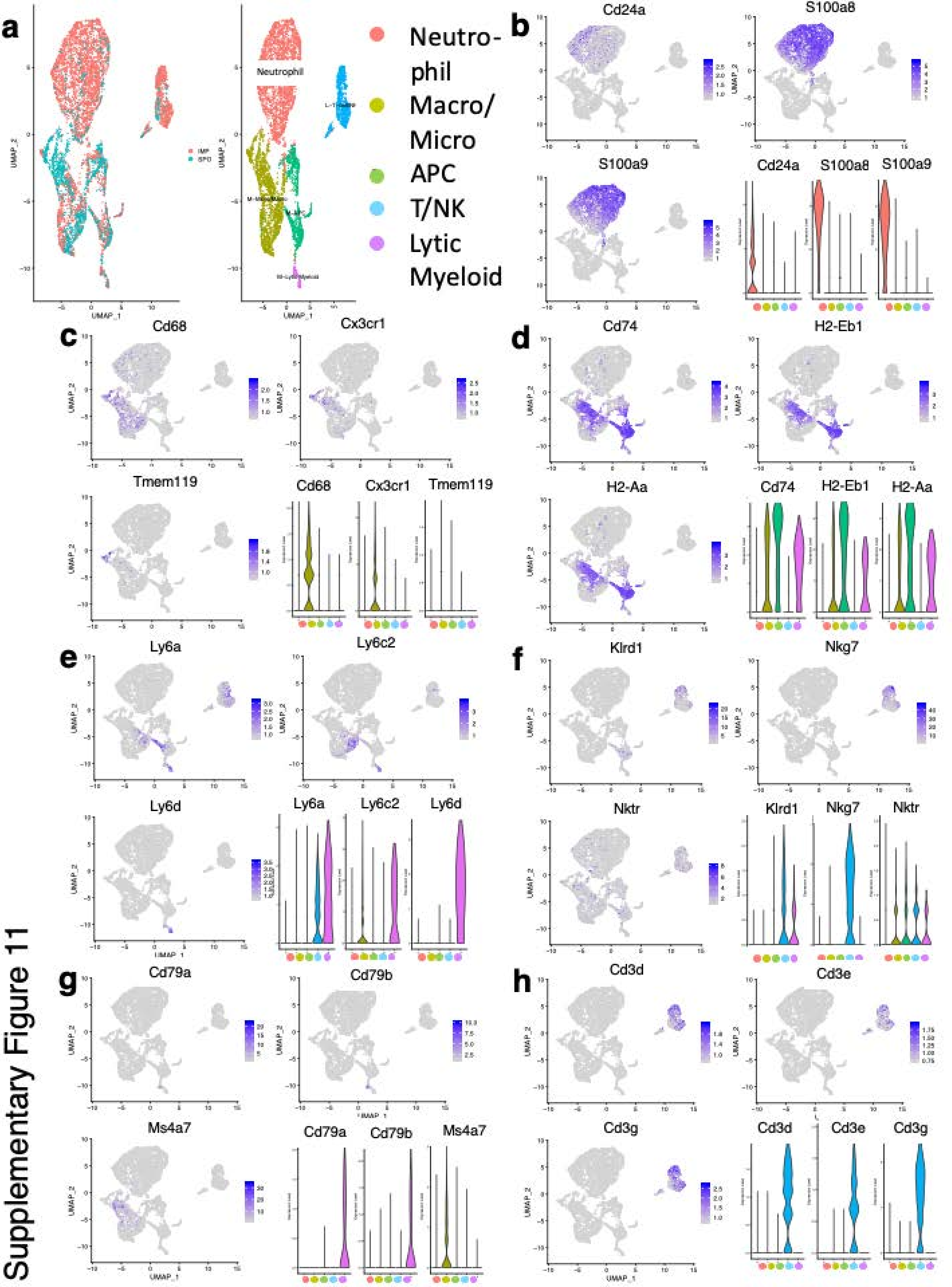

Supplementary Figure 11

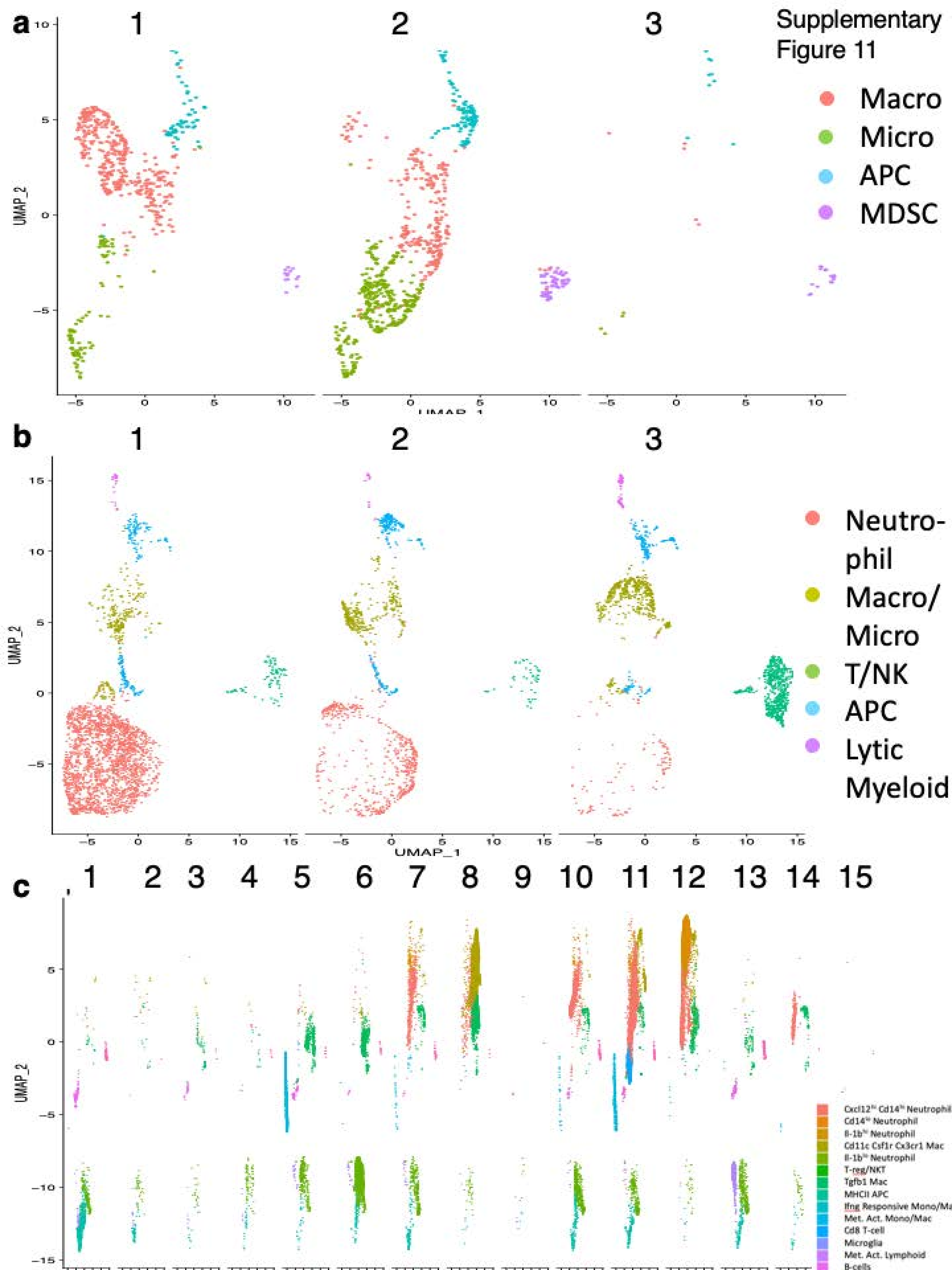
Supplementary Figure 11
a
1
2
3
Macro
Micro
APC
MDSC
UMAP_2
UMAP_1
b
1
2
3
Neutro-phil
Macro/Micro
T/NK
APC
Lytic Myeloid
UMAP_2
UMAP_1
c
1
2
3
4
5
6
7
8
9
10
11
12
13
14
15
UMAP_2
Cxcl12 Cd14 Neutrophil
Cd14 Neutrophil
Il-1b Neutrophil
Cd11c Csf1r Cx3cr1 Mac
Il-1b Neutrophil
T-reg/NKT
Tgfb1 Mac
MHCII APC
Ifng Responsive Mono/Mac
Met. Act. Mono/Mac
Cd8 T-cell
Microglia
Met. Act. Lymphoid
B-cells

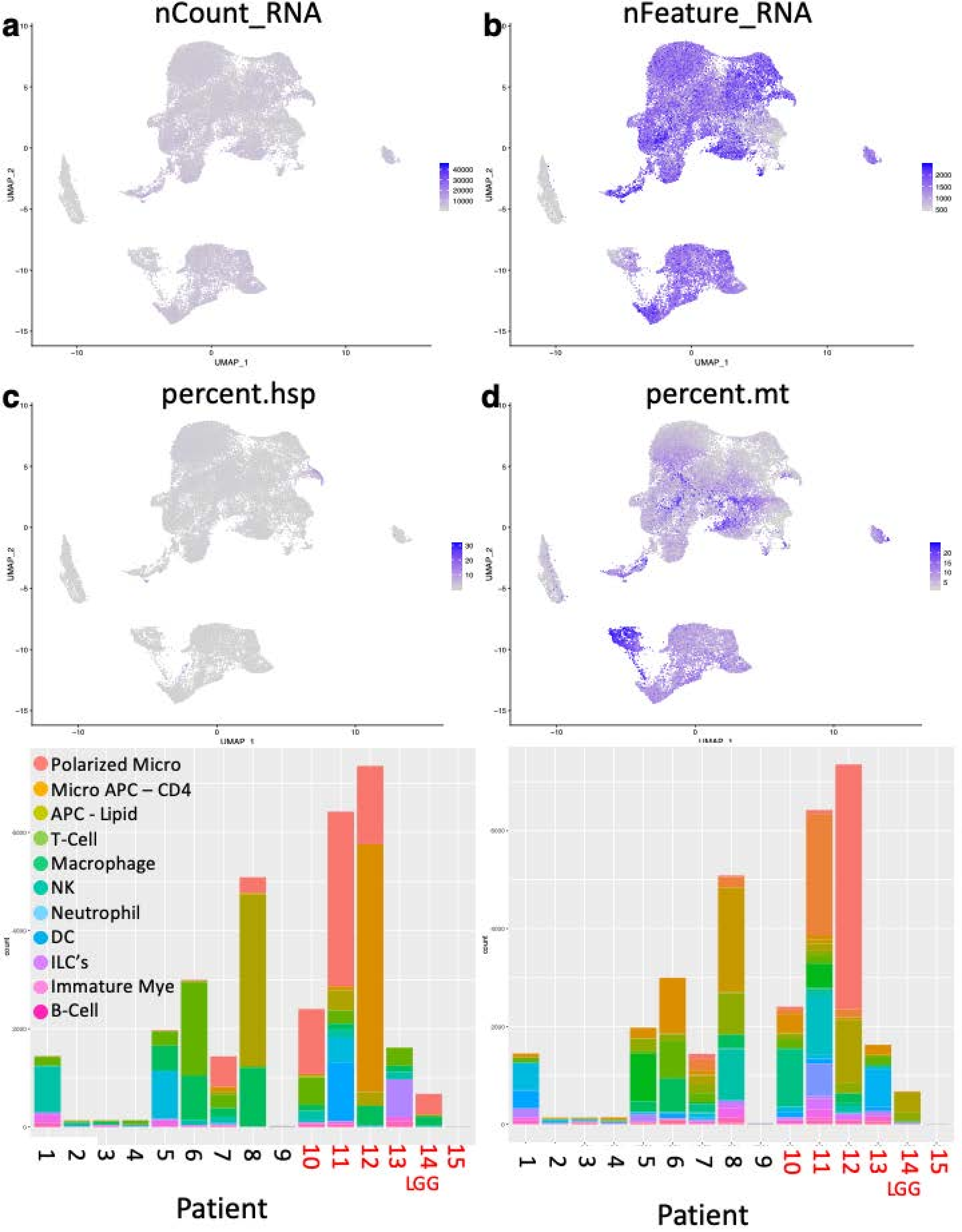

Supplementary Figure 13

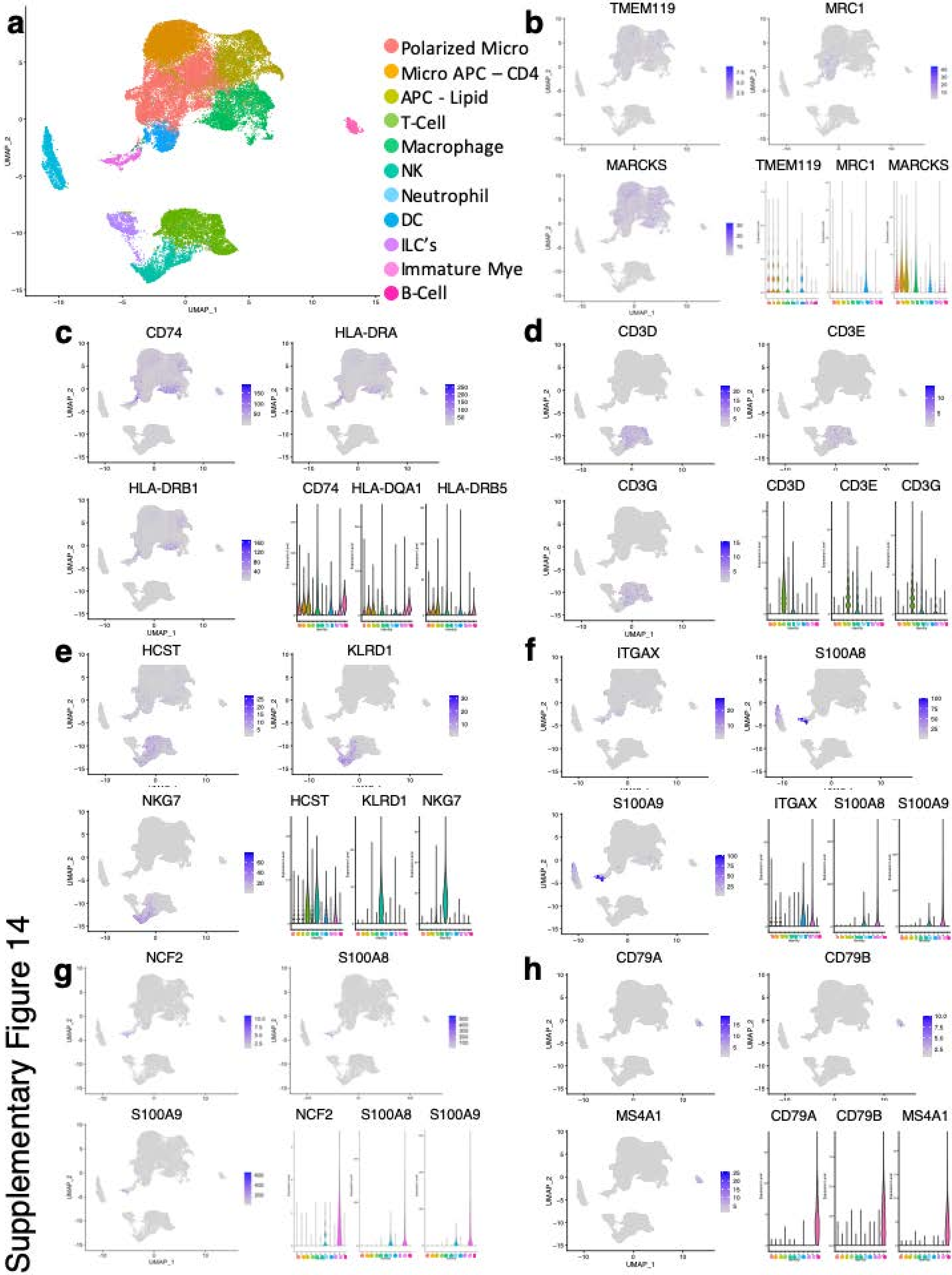

Supplementary Figure 14

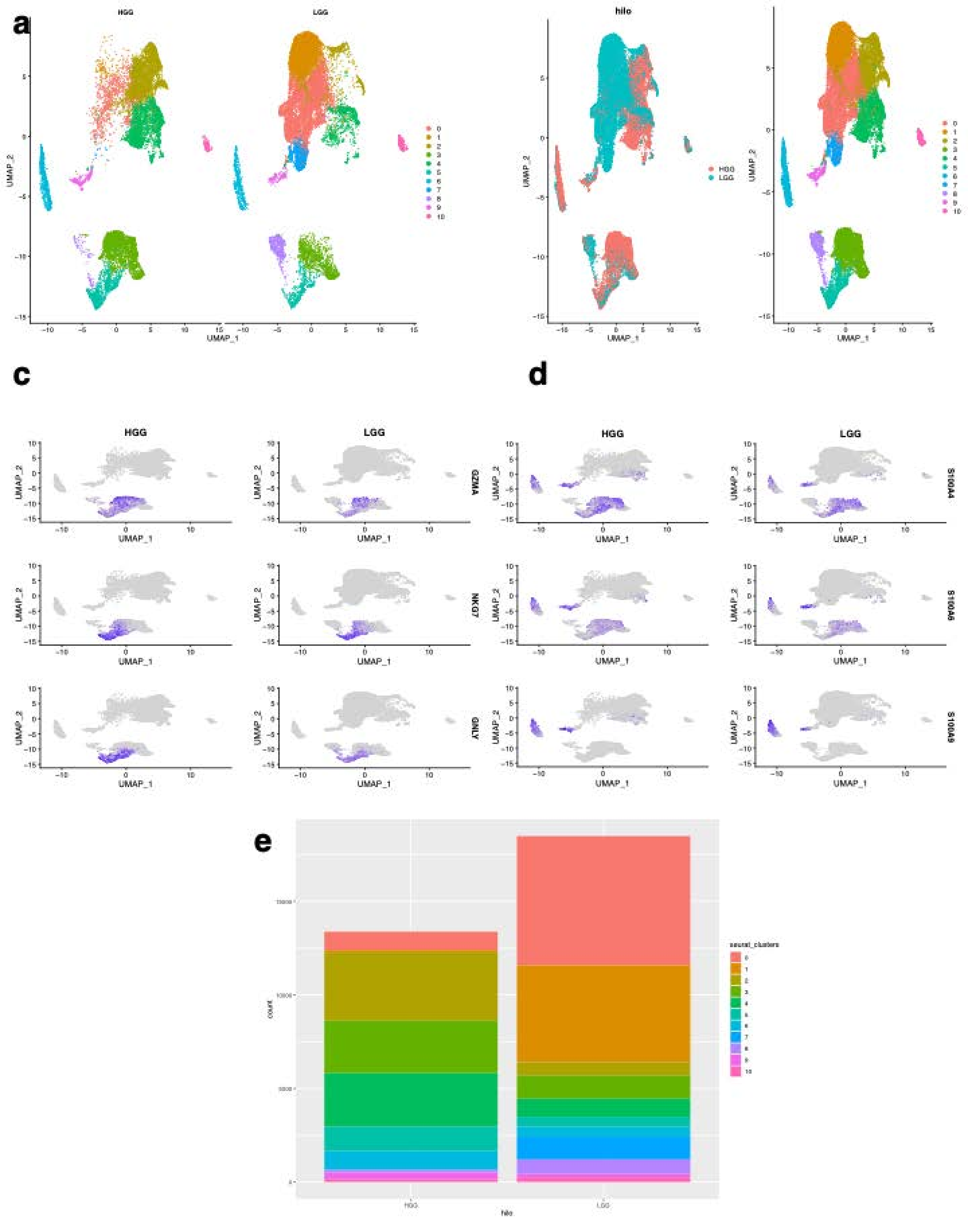

Supplementary Figure 15
